# Serial Passage of *Cryptococcus neoformans* in *Galleria mellonella* Results in Increased Capsule and Intracellular Replication in Hemocytes, but Not Increased Resistance to Hydrogen Peroxide

**DOI:** 10.3390/pathogens9090732

**Published:** 2020-09-05

**Authors:** Muhammad Fariz Ali, Stephen M. Tansie, John R. Shahan, Rebecca L. Seipelt-Thiemann, Erin E. McClelland

**Affiliations:** 1 Department of Biology, Middle Tennessee State University, Box 60, 1301 E. Main Street, Murfreesboro, TN 37132, USA; mali17@uthsc.edu (M.F.A.); smt0066@auburn.edu (S.M.T.); j.richard.shahan@gmail.com (J.R.S.); Rebecca.Seipelt@mtsu.edu (R.L.S.-T.); 2 M&P Associates, Inc., P.O. Box 11534, Murfreesboro, TN 37129, USA

**Keywords:** *C. neoformans*, virulence, adaptation, *G. mellonella*, hydrogen peroxide

## Abstract

To gain insight into how pathogens adapt to new hosts, *Cryptococcus neoformans* (H99W) was serially passaged in *Galleria mellonella*. The phenotypic characteristics of the passaged strain (P15) and H99W were evaluated. P15 grew faster in hemolymph than H99W, in vitro and in vivo, suggesting that adaptation had occurred. However, P15 was more susceptible to hydrogen peroxide in vitro, killed fewer mouse macrophages, and had less fungal burden in human ex vivo macrophages than H99W. Analysis of gene expression changes during *Galleria* infection showed only a few different genes involved in the reactive oxygen species response. As P15 sheds more GXM than H99W, P15 may have adapted by downregulating hemocyte hydrogen peroxide production, possibly through increased capsular glucuronoxylomannan (GXM) shedding. Hemocytes infected with P15 produced less hydrogen peroxide, and hydrogen peroxide production in response to GXM-shedding mutants was correlated with shed GXM. Histopathological examination of infected larvae showed increased numbers and sizes of immune nodules for P15 compared to H99W, suggesting an enhanced, but functionally defective, response to P15. These results could explain why this infection model does not always correlate with murine models. Overall, *C. neoformans’* serial passage in *G. mellonella* resulted in a better understanding of how this yeast evolves under selection.

## 1. Introduction

*Cryptococcus neoformans* is an opportunistic pathogen that is able to infect and survive long term in many different species. To accomplish extended survival, even in healthy hosts, it resides and replicates in macrophages and other macrophage-like immune cells. To gain additional insight into the pathways involved in intracellular replication of *C. neoformans*, the serotype A strain H99W [1] was serially passaged in the wax moth larvae *Galleria mellonella* for 100 *C. neoformans* generations (15 passages). *Galleria mellonella* is an increasingly useful invertebrate host for *C. neoformans*. The waxworms are inexpensive, easy to work with, and contain phagocytic hemocytes which, similarly to mammalian macrophages and neutrophils, phagocytose and kill *C. neoformans* [2], likely through the production of high levels of reactive oxygen species (ROS) [3]. Additionally, numerous *C. neoformans* virulence factors are important in both *G. mellonella*, as well as mammalian infections [2], particularly the capsule, which has been shown to have numerous effects on the host immune response [4]. We hypothesized that serial passage of *C. neoformans* in *G. mellonella* would result in a strain that would be adapted to the larval environment with selection pressures provided by the organismal environment and various aspects of the larval immune response, and thus be more resistant to ROS and/or have a higher level of intracellular replication.

To test these ideas, several experiments were undertaken including comparisons between the parent and adapted strain in in vitro growth medium containing *Galleria* hemolymph, fungal burden in *Galleria* larvae, fungal burden and killing in mouse and human macrophages, differential gene expression during infection in *Galleria* larvae, in vitro susceptibility to hydrogen peroxide, hydrogen peroxide production from *Galleria* hemocytes during infection, and histological features of the larval immune response.

Overall, these data suggested that strain P15′s adaptation involved reducing the hemocyte ROS response by increasing capsule shedding without blocking the entire immune response. However, this adaptation was not generally successful across species, as it was not protective in mouse or human macrophages. Thus, the use of serial passage experiments has led to an increased understanding of how *C. neoformans* interacts with the *G. mellonella* immune response and illustrates how it evades and/or disables specific components of the *G. mellonella* ROS. These data have resulted in a better understanding of how this yeast evolves under selection and illustrates that, similar to in mice and humans [4], capsule is a potent immune modulator in *G. mellonella*.

## 2. Results

**Figure 1:**
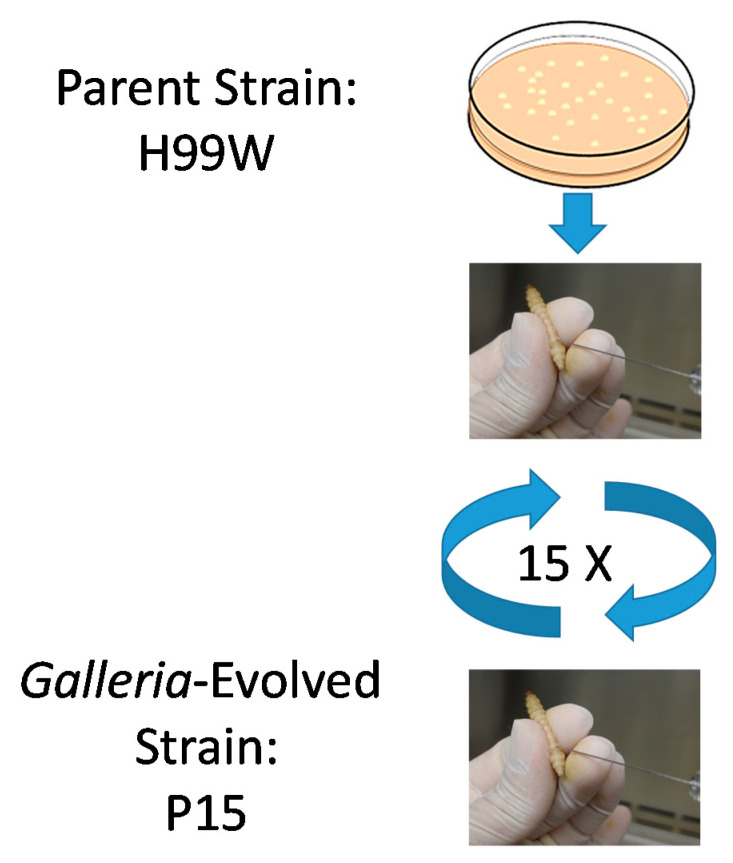
*Galleria*-adapted strain generation. *Cryptococcus neoformans* strain H99W was serially passaged in *Galleria mellonella* larvae 15 times to develop the adapted strain P15. One thousand *C. neoformans* cells were used to infect 15 larvae for each three-day passage. On the third day, hemolymph was collected from all remaining larvae, pooled, counted and used to infect the next passage. Remaining hemolymph was frozen and stored at −80 °C.

To generate a *Galleria*-adapted strain of *C. neoformans*, a primary culture of strain H99W, which is a less virulent strain compared to another common reference strain H99S [1], was grown from a frozen stock and used to infect *Galleria* larvae. After three days, hemolymph was collected, pooled, and used to inoculate additional *Galleria* larvae. This process was repeated, going directly from larvae to larvae a total of fifteen times, which was equivalent to approximately 100 fungal generations, to generate the putative *Galleria*-adapted strain, which was termed P15 (passage 15) (Figure 1).

### 2.1. Evidence for Adaptation

**Figure 2:**
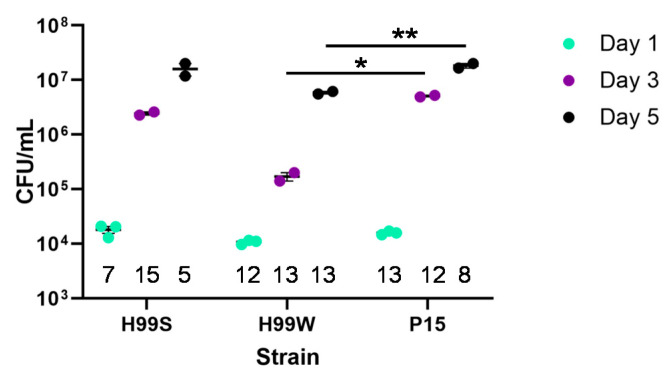
In vivo fungal burden of the *Galleria*-adapted strain P15, the parent strain H99W, and the virulent strain H99S. Fifteen *Galleria* larvae were infected with 2000 cells of each strain for each time point. Fungal burden was quantified from hemolymph collected from 5–15 larvae at days 1, 3, and 5 and normalized to the number of larvae collected at each day. Data are numbers from two separate yeast peptone dextrose plates for each time point. Error is standard error of the mean. The data are representative of three independent experiments. Numbers along the bottom represent the sample size. * *p* = 0.019, ** *p* < 0.0001, analysis of variance with simple contrasts. CFU, colony forming units.

If strain P15 had adapted to the *Galleria* environment, it would be expected to have acquired characteristics not found in the parent strain, including enhanced growth and survival in the adaptive environment. To determine if strain P15 had adapted to the *Galleria* environment, doubling times were evaluated in vitro in minimal medium with hemolymph. In vivo growth of each strain (fungal burden, colony forming units (CFU)) was also assessed during the course of infection in *Galleria*. P15 grew faster in hemolymph-containing medium in vitro than the parent strain H99W and the more virulent reference strain H99S (Table 1) and showed significantly higher CFU at days 3 and 5 post-infection than the parent strain H99W (Figure 2, *p* < 0.001 and *p* < 0.0001, respectively). In addition, P15 had significantly higher CFU at day 5 compared to the more virulent reference strain H99S (Figure 2, *p* < 0.0001).

**Figure 3:**
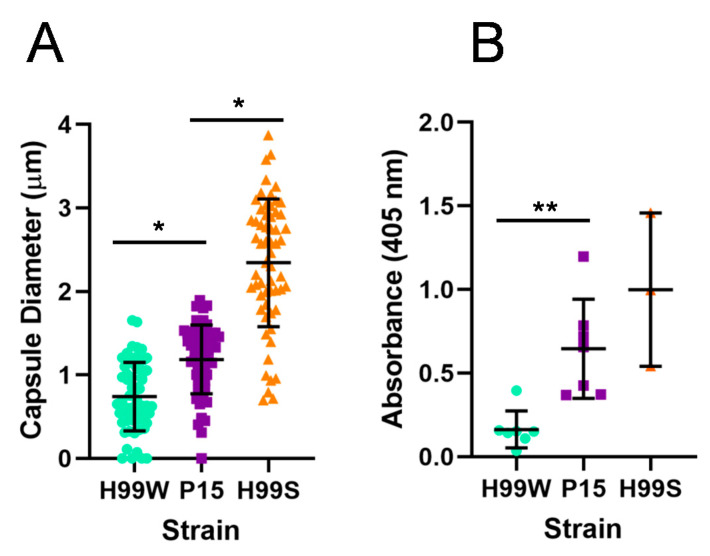
Capsule diameter and shedding of the pre-passage H99W, P15, and the virulent H99S strains. Graphs showing capsule size (**A**) and capsule shedding (**B**) for strains H99W, the *Galleria*-adapted strain P15, and the virulent H99S strain for comparison. Cell body diameter and capsule diameter were measured using the Axiovision software (Carl Zeiss Microscopy, LLC, White Plains, NY, USA). Capsule diameter was calculated by subtracting the cell body diameter from the diameter of the entire cell + capsule and dividing by two. For capsule size, the mean and standard deviation of two independent experiments are depicted. Sample size is 30 cells per strain. * *p* < 0.05, analysis of variance with Tukey’s honestly significant difference test. For capsule shedding, the mean and standard deviation of three independent experiments are depicted. ** *p* = 0.0017, analysis of variance. Sample sizes are 7, 7 and 3 for H99W, P15 and H99S, respectively.

One of the major virulence factors for *C. neoformans* is its large complex capsule, which is known to protect the yeast from the host immune response [5,6]. In addition, shed capsule is known to be immunomodulatory [4]. To determine whether this virulence characteristic had changed during the process of adaptation, capsule size and amount of capsule shed by P15, H99W, and H99S strains were measured. As expected, P15 had a significantly larger capsule (Figure 3A) and also shed significantly more capsule than the parent strain (H99W), but had a smaller capsule and shed less than the more virulent reference strain (H99S) (Figure 3B).

**Figure 4:**
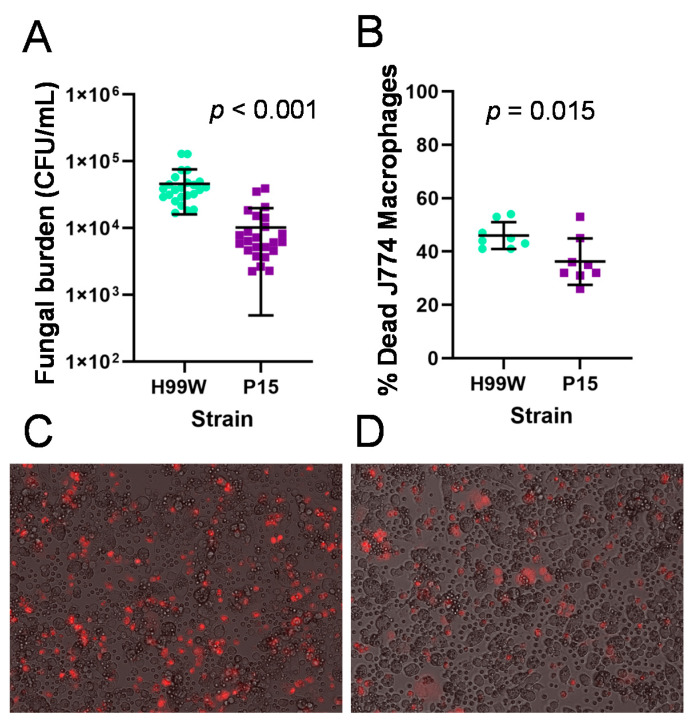
Fungal burden and macrophage death of J774.16 macrophages infected with the parent strain H99W and the *Galleria*-adapted strain P15. (**A**). Fungal burden in human ex vivo macrophages. The mean and standard deviation are depicted showing decreased fungal burden in human ex vivo macrophages incubated for 18 h with strain P15 compared to the pre-passage parent strain H99W. The graph depicts data from two independent experiments that used macrophages from two different donors for each experiment. Sample size is 24 counts for each strain. (**B**). Strain P15 kills less J774.16 macrophages. The mean and standard deviation are depicted showing decreased killing of J774.16 macrophages by strain P15 after incubation for 24 h compared to the pre-passage parent strain H99W. The graph depicts data from two independent experiments. Sample size is eight wells for each strain. Images of J774.16 cells infected with H99W (**C**) or P15 (**D**). Red stain is propidium iodide. Images were captured with a Nikon (Nikon, Tokyo, Japan) Eclipse Ti inverted fluorescent microscope at 15× magnification.

To investigate host-pathogen differences between the *Galleria*-adapted strain and its parent strain in other well-characterized hosts of *C. neoformans*, the ability of each strain to survive and replicate within mouse and ex vivo human macrophages was examined. Based on the growth and capsule data, it was expected that the adapted strain would survive better and replicate to higher levels in macrophages, as well as kill macrophages more often than the parent strain. However, the pre-passage H99W strain and the P15 strain showed no difference in intracellular replication within J774.16 and RAW264.7 mouse macrophages (data not shown). Surprisingly, strain P15 showed significantly less fungal burden after 18 h of incubation with ex vivo human macrophages (Figure 4A, *p* < 0.001) and killed fewer J774.16 mouse macrophages after 24 h incubation than H99W (Figure 4B).

### 2.2. Identifying a Putative Adaptation Mechanism

To gain insight into the unexpected and conflicting effects on host cell survival, studies to compare the gene expression responses of both strains in the *Galleria* environment were undertaken using microarray analysis. Microarray analysis showed 172 genes were upregulated and 254 genes were downregulated in strain P15 compared to the parent H99W strain (Supplementary Table S1). In total, these genes represented fourteen different molecular functions, processes, or components, including cell cycle, cell wall, energy, housekeeping, metabolism, pathogenicity, protein degradation, protein integrity, secretion, signal transduction, stress response, transcription, translation, and unknown, as determined by National Center for Biotechnology Information (NCBI) Gene Ontology Tools (Bethesda, MD, USA). While nearly all classifications included both upregulated and downregulated genes, only downregulated genes were found in the signal transduction classification. To further investigate the molecular function of these genes and their possible roles in adaptation, the Kyoto Encyclopedia of Genes and Genomes database pathway tool (Kanehisa Laboratories, Kyoto, Japan) was used to determine which functional pathways were affected based on these differentially expressed genes. Genes involving the spliceosome, ribosome, homologous recombination, nucleotide excision repair and the ubiquitin-mediated proteolytic pathways were upregulated in strain P15 compared to H99W, while genes involved in various metabolic pathways, RNA transport and degradation, basal transcription, biotin and sulfur metabolism, and peroxisome biogenesis pathways were down-regulated in strain P15 compared to strain H99W.

**Figure 5:**
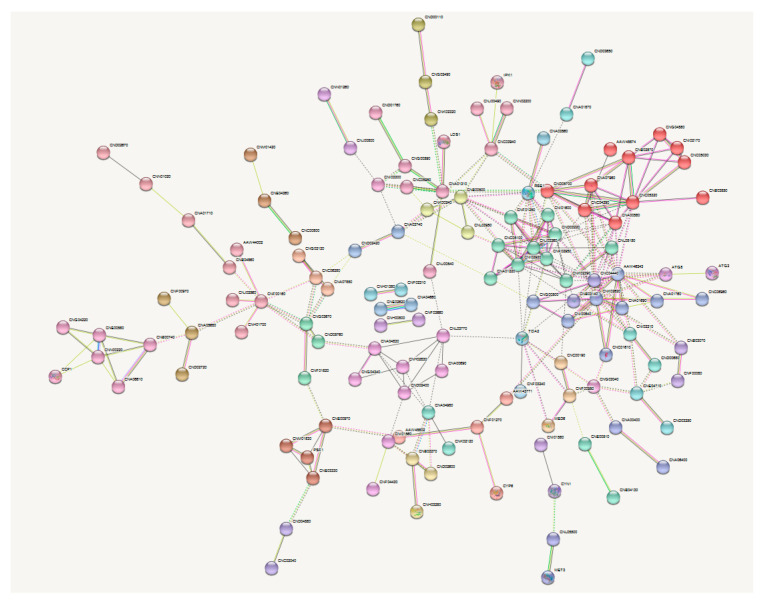
Protein interaction network of differentially expressed genes. The Search Tool for the Retrieval of Interacting Genes/Proteins (STRING) database was used to identify protein-protein interaction (PPI) networks among the differentially expressed genes. While no significant differences were found within the dataset, the number of PPIs within the dataset was significant (*p* = 2.24 × 10^−5^), indicating biological significance. Unconnected nodes are not shown and data is clustered using the Markov Clustering Algorithm, MCL.

To determine if these genes had significant protein interactions, the protein interaction networks among the 400 differentially expressed genes identified by microarray analysis were investigated using the Search Tool for the Retrieval of Interacting Genes/Proteins (STRING, version 11.0, ELIXIR, Cambridgeshire, UK) database. A large proportion of proteins encoded by these genes were not identified to have interactions within the dataset. Based on the dataset size, 198 interactions would be expected, but more were identified with a protein-protein interaction (PPI)-enrichment *p*-value of 2.24 × 10^−5^, indicating the data pool was likely to have biological significance (Figure 5). However, no significant clusters were found.

To confirm the microarray results, 36 genes were tested using quantitative reverse transcription-polymerase chain reaction (qRT-PCR; Supplementary Table S2). The genes were chosen based on the highest fold change for each functional classification and/or the gene ontology description. Strikingly, 94% of eighteen genes upregulated in strain P15 compared to strain H99W were verified using qRT-PCR, whereas only 33% of eighteen genes downregulated in strain P15 compared to strain H99W were verified (Supplementary Table S2).

A number of genes are thought to be involved in *C. neoformans* resistance to oxidative damage, including genes encoding glutathione peroxidase (*Gpx1* and *Gpx2*), peroxiredoxin (*Tsa1*), cytosolic superoxide dismutase (*Sod1*), mitochondrial superoxide dismutase (*Sod2*), and the alternative oxidase (*Aox1*) [7]. Two of these genes were identified as differentially expressed in the microarray analysis and were verified using qRT-PCR. Expression of only a single ROS-related gene, glutathione peroxidase (*Gpx2*, CNN00220), was upregulated in strain P15 compared to H99W (5.7 fold; average of two qRT-PCR experiments). Most ROS-related genes were not differentially expressed, including peroxiredoxin (*Tsa1*), which is a thiol peroxidase known to provide defense against oxidative and nitrosative damage and is induced in the presence of hydrogen peroxide in vitro [8]. In addition, neither of the mRNAs encoding the cytosolic and mitochondrial forms of superoxide dismutase (Sod1 and Sod2) enzymes, required to convert superoxide to hydrogen peroxide and water [9], were differentially expressed. Finally, alternative oxidase (encoded by *Aox1*), an enzyme thought to be involved in resistance to ROS, which is induced upon exposure to superoxide anion [10], was downregulated 1.2 fold (average of two qRT-PCR experiments) in strain P15 (Supplementary Table S2), suggesting that strain P15 was not exposed to ROS during *Galleria* infection, and that P15 had survived based on a different mechanism than increased tolerance to ROS.

### 2.3. Validating the Putative Adaptation Mechanism

**Figure 6:**
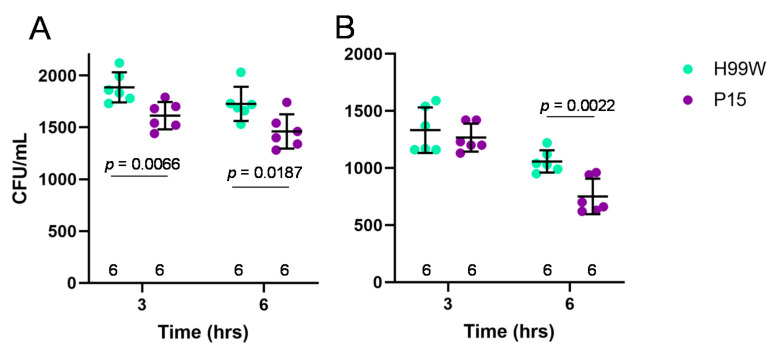
Fungal burden of the parent strain H99W and the adapted strain P15 in the presence of oxidative stress. (**A**). The mean and standard deviation are depicted showing that strain P15 is significantly more susceptible to 0.5 mM hydrogen peroxide than H99W at both three and six h of incubation. The graph depicts data from two independent experiments. Numbers at the bottom represent the sample size. (**B**). The mean and standard deviation are depicted showing increased susceptibility of strain P15 with induced capsule to 0.5 mM hydrogen peroxide after six h of incubation compared to strain H99W with induced capsule. The graph depicts data from two independent experiments. Numbers at the bottom represent the sample size. Significance was tested using analysis of variance with simple contrasts. CFU: colony forming units.

Based on the lack of an oxidative stress response in P15-infected hemocytes at the transcriptome level and the ability for human and mouse macrophages to kill strain P15 more than its parent strain, the *Galleria*-adapted strain would be expected to have a distinctly lower tolerance to oxidative and nitrosative exposure compared to the non-adapted parent strain. To determine P15′s resistance to ROS or reactive nitrogen species (RNS), fungal survival after exposure to hydrogen peroxide or sodium nitrite + succinic acid was measured. Incubation with hydrogen peroxide exposes the yeast to reactive OH groups via the Fenton reaction [11], while incubation with sodium nitrite + succinic acid exposes the yeast to reactive NO groups [12]. As expected, strain P15 was significantly more susceptible to cell death whether it was incubated for three (*p* = 0.006) or six (*p* = 0.0187) h with 0.5 mM hydrogen peroxide (Figure 6A). To determine if capsule could induce protection from ROS, the experiment was repeated following capsule induction for both H99W and strain P15. Strain P15 was again significantly more susceptible to damage by 0.5 mM hydrogen peroxide, but only after exposure for six h (*p* = 0.0022, Figure 6B). There was no difference in susceptibility to sodium nitrite + succinic acid between strains H99W and P15 (data not shown).

**Figure 7:**
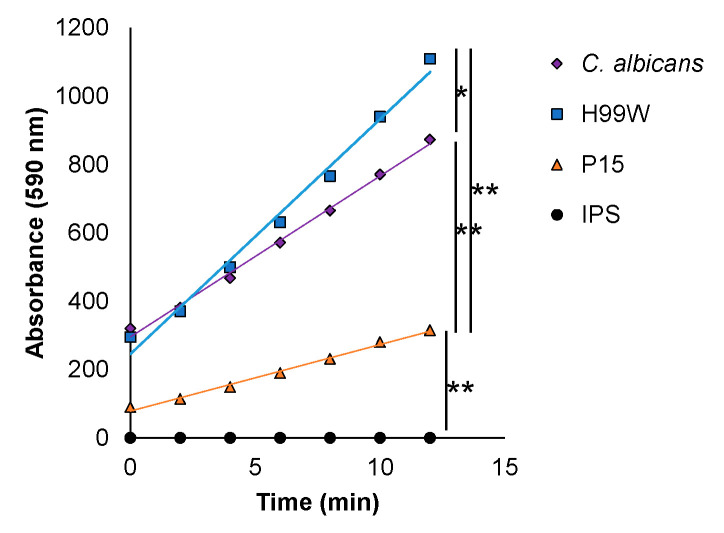
Hydrogen peroxide production in hemocytes of *Galleria* infected with the parent strain H99W, the adapted strain P15, and *C. albicans*. Ten larvae were infected with 2000 cells. Hemocytes were collected, pooled by strain, and assayed for hydrogen peroxide production at 24 h post-infection using a fluorescent assay. IPS = insect physiological saline. Lines are best fit linear regression lines where the slopes of the lines are compared using Tukey’s HSD for all pairwise comparisons. Data are the average of three independent experiments. * *p* = 0.002, ** *p* < 0.0001.

The results showing that P15 exhibited only a subset of the expected oxidative stress and damage responses compared to the parent strain, as well as showing reduced resistance to peroxide in vitro and a reduced ability to kill mouse macrophages, suggested that perhaps P15 had adapted to block or otherwise negate *Galleria*’s oxidative response rather than withstand it, as was initially hypothesized. To address this possibility, hemocyte hydrogen peroxide production from *Galleria* larvae that had been infected with each strain was compared. Hemocytes from P15-infected larvae were able to produce hydrogen peroxide, although to a significantly lesser extent than hemocytes from either H99W-infected larvae or *Candida*-infected larvae (Figure 7), which supported a mechanism in which strain P15 reduced the host response rather than resisting it.

**Figure 8:**
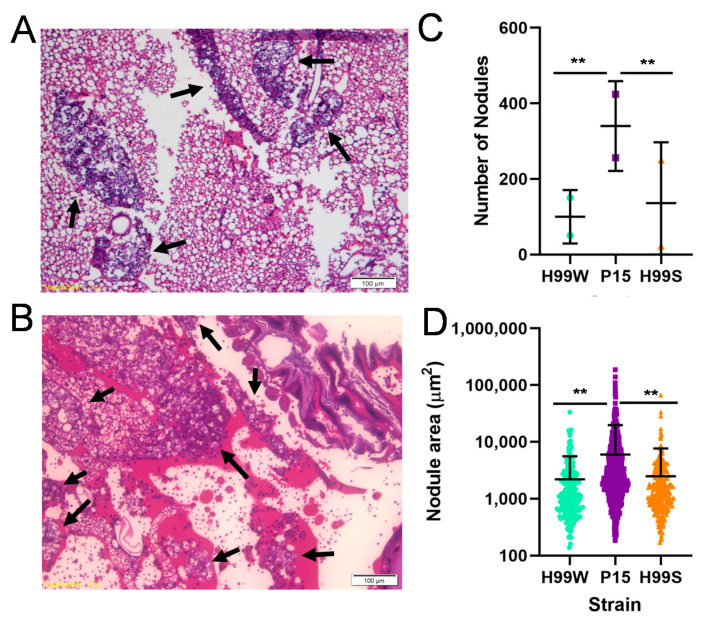
Immune response of *Galleria* infected with the parent strain H99W, the adapted strain P15, and the virulent H99S strain. Two larvae were infected with 3000 cells of either H99W, P15, or H99S and incubated for five days. Larvae were then fixed, bisected, sectioned, and stained with hematoxylin and eosin. Nodule number and size were counted/measured using the center-most section of each larva. A representative image for H99W (**A**) and P15 (**B**) are shown. Black arrows indicate nodules. Small purple dots in (**B**) within white spaces are P15 not enclosed within nodules. Comparison of the nodule number and nodule size for each strain is shown in (**C**) and (**D**), respectively. The mean and standard deviation of the number of nodules per larva are depicted (**C**). Significance was tested using Χ^2^, ** *p* < 0.0001. The mean and standard deviation of the size of the nodules are depicted for both larvae (**D**). Significance was tested using the nonparametric Wilcoxon Rank Sums test, ** *p* < 0.0001.

To not only investigate whether *Galleria* larvae were mounting any immune response to infection, but also to gain a broader perspective of how the *Galleria* host’s immune response differed for the adapted strain, the organismal immune response to infection with each strain was examined using histological techniques. The number and size of immune nodules are known to be good indicators of the *Galleria* immune response [13]. Larvae that had been infected with each strain for two and five days were fixed, sectioned and stained. The number and size of immune nodules surrounding *C. neoformans* cells were then measured. Larvae infected with P15 had significantly more and larger nodules than those infected with H99W at day 5 (Figure 8C,D). In addition, there was a large amount of P15 that was not enclosed within nodules (Figure 8B).

### 2.4. Experimental Evidence for Mechanism

**Figure 9:**
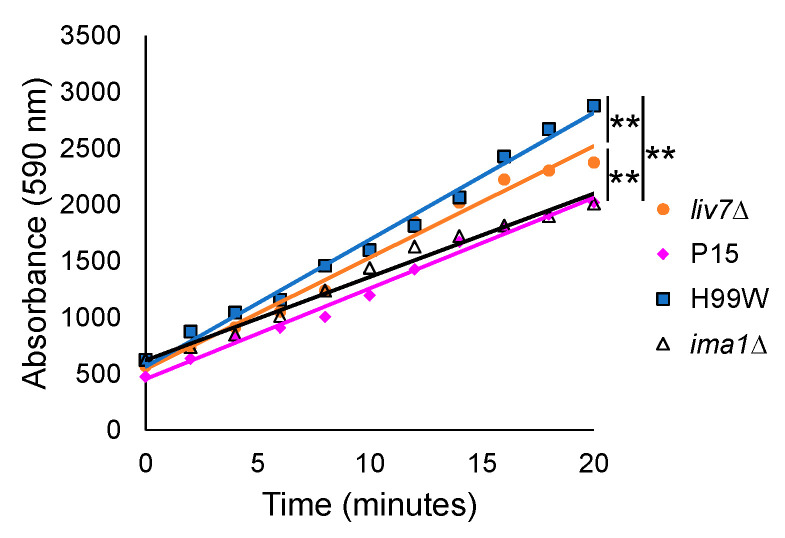
Hydrogen peroxide production from hemocytes infected with glucuronoxylomannan (GXM) secretion mutants (*liv7*∆ and *ima1*∆) of *C. neoformans*. Ten larvae were infected with 2000 cells. Hemocytes were collected, pooled by strain, and assayed for hydrogen peroxide production at 24 h post-infection using a fluorescent assay. Lines are best fit linear regression lines. The slopes of the lines are compared using Tukey’s HSD for all pairwise comparisons. Data shown are one representative experiment. The experiment was repeated three times. ** *p* < 0.0001.

The observations that (1) *Galleria* larvae exhibited an immune response to strain P15, (2) P15-infected hemocytes produced less hydrogen peroxide in response to infection, and (3) the P15 strain had a larger capsule and shed more capsule than H99W, suggested that strain P15′s ability to depress *Galleria*’s ROS response, but not the entire immune response, might be due to its increased capsule shedding. To test this hypothesis, *Galleria* larvae were infected with GXM secretion mutants, strains that exhibit decreased capsule shedding, *liv7Δ* (CNAG_06464, [14]) or increased GXM capsule shedding, *ima1Δ* (CNAG_00658, [14]). Hemocytes isolated from infected larvae were then tested for hydrogen peroxide production. Hemocytes from *Galleria* infected with *liv7Δ* showed increased production of hydrogen peroxide compared to P15 and decreased production of hydrogen peroxide compared to H99W (Figure 9). In comparison, hemocytes from *Galleria* infected with *ima1Δ* produced approximately the same amount of hydrogen peroxide as those infected with P15, which was significantly less than hemocytes infected with H99W (Figure 9).

## 3. Discussion

A number of invertebrate and non-mammalian vertebrate host models, such as zebrafish [15,16], worms [17,18], flies [19,20], amoebae [21,22] and moths [2,23], have been used to understand host-microbial interactions. These models have numerous benefits, such as reduced cost, ease of handling, less limitations on use, a more straightforward system, and often better genetic tools. Thus, these kinds of host models contribute greatly to understanding microbial pathogenesis. Serial passage of the pathogen, *C. neoformans*, through a distinct host environment, *Galleria mellonella* larvae, should result in adaptation of the pathogen to that environment. Since the passaged strain P15 showed increased growth in vitro in hemolymph-containing medium and had increased fungal burden in vivo at days 3 and 5, strain P15 successfully adapted to the *Galleria* environment compared to the parent strain, H99W. Our original hypothesis was that the adapted strain would increase in virulence after passage because it is known that serial passage within a host increases virulence [24]. We hypothesized that the increased virulence would be via an increased resistance to *Galleria* ROS, resulting in a higher level of intracellular replication. To begin to understand the process and mechanism of adaptation, virulence-related phenotypes were initially examined in P15 compared to the parent strain and the more virulent H99S strain, including capsule size and capsule shedding. As expected, the adapted strain had a larger capsule and also shed more capsule than the parent strain, consistent with its more virulent phenotype in *Galleria* larvae. However, while strain P15 showed indications of adaptation to the *Galleria* hemolymph environment, it did not show more or even equivalently virulent phenotypes in subsequent experiments. For example, P15 did not show the hypothesized increase in in vitro intracellular replication in mouse macrophages compared to the parent strain. Furthermore, strain P15 actually showed decreased survival in ex vivo human macrophages and a reduced ability to kill mouse macrophages in vitro.

To identify the molecular pathways responsible for these incongruent results, differential gene expression analyses of RNA from each strain grown in *Galleria* was undertaken by microarray analysis. Of the ~6500 genes in the *C. neoformans* genome, only 426 were changed between strains P15 and H99W. Of the 254 genes that were downregulated in strain P15 compared to strain H99W, the most downregulated genes were involved in metabolic processes and synthesis of secondary metabolites. The most upregulated genes were involved in basic cellular processes that can be regulated, particularly in a general stress response, such as the ribosome, spliceosome, and proteolysis [25]. However, in the general stress response, these genes are usually downregulated, not upregulated. The lack of evidence for either a general or specific stress response, particularly since both hemocytes and macrophages are known to produce hydrogen peroxide to combat and destroy fungi, was surprising [26,27]. Since both mammalian macrophages and hemocytes are known to kill fungal pathogens using similar mechanisms that involve ROS [26,28], genes involved in preventing oxidative damage would be expected to be upregulated in P15 compared to the parent strain. In a closer examination of the differentially expressed genes, only one gene involved in the host’s response via ROS was upregulated. All other genes related to ROS generation were either not significantly different or down-regulated. To identify any significant protein-protein interactions, as well as a network of interacting genes, the search tool for the retrieval of interacting genes/proteins (STRING) database was used. While the initial group of proteins had significantly more interactions and were biologically connected, deeper investigation did not reveal any significant protein clusters.

Taken together, these results suggested that strain P15′s adaptation to *Galleria* did not involve development of resistance to the host’s ROS response, but rather involved development of the ability to specifically subvert initiation of the ROS response in *Galleria*, but not in mouse or human macrophages. To confirm the suspected, continued sensitivity of P15 to ROS and RNS, in vitro sensitivity to hydrogen peroxide and reactive nitrogen was measured. Incubation with hydrogen peroxide exposes the yeast to reactive OH groups via the Fenton reaction [11], while incubation with sodium nitrite + succinic acid exposes the yeast to reactive NO groups [12]. Strain P15 was more susceptible to hydrogen peroxide than strain H99W at both 3 and 6 h of incubation. Since this result could be due to lack of a capsule, capsule was induced and the experiment was repeated. Again, strain P15 was more susceptible to hydrogen peroxide, but this time only after 6 h, suggesting that, as shown previously, the capsule protects the yeast against damage by ROS [29].

Along with continued sensitivity to hydrogen peroxide in vitro, this model of adaptation predicted that *Galleria* infected with strain P15 would produce less hydrogen peroxide than those infected with the parent strain. Indeed, hemocytes isolated from *Galleria* larvae infected with P15 produced significantly less hydrogen peroxide than those infected with either the parent strain H99W or the positive control, *Candida albicans*. However, P15 does not, in fact, have the ability to depress the entire immune response, as histological studies of larvae infected for two and five days indicate P15-infected larvae have significantly more and larger immune nodules at day 5, indicating that *Galleria* actually have an enhanced immune response to P15 compared to H99W, but are unable to effectively produce ROS [13], compared to larvae infected with the parent strain.

Finally, to gain a clearer understanding of how strain P15, which has a larger capsule and higher levels of capsule shedding than its parent strain, might reduce the *Galleria* ROS response, we examined the ability of *C. neoformans* mutant strains with different capsule shedding phenotypes to induce hydrogen peroxide production from hemocytes. Hemocytes from larvae infected with the *liv7*∆ mutant, which sheds two-fold less capsule than H99S, induced more hydrogen peroxide than those infected with P15, while hemocytes from larvae infected with the *ima1*∆ mutant, which sheds five- to ten-fold more capsule than H99S, induced less hydrogen peroxide than the parent strain, but equivalent amounts to P15. Both the *liv7*∆ and *ima1*∆ mutants have wildtype levels of surface-associated capsule [14], suggesting that the amount of shed GXM was affecting hemocyte production of ROS.

In summary, these studies began with the hypothesis that a serially passaged strain, P15, would show increased resistance to ROS compared to its parent strain. However, strain P15 showed increased susceptibility to hydrogen peroxide in vitro, which was unexpected, as 100 generations of exposure to ROS may have increased the strain’s resistance or defense against *Galleria* ROS. However, these data suggest that the fungus utilized another mechanism to adapt, by depressing the host’s ability to produce ROS, but not the host’s entire immune response. Furthermore, experiments using *C. neoformans* mutants with variation in capsule shedding phenotypes provided further support that the mechanism P15 utilized in adapting to *Galleria* was to increase capsule shedding, which allowed increased survival due to a reduction in hemocyte ROS production. However, this adaptation did not have the same effect on mouse or human macrophage ROS production, which is surprising given that the amount of shed GXM has been correlated with differences in virulence in mice [30] and humans [31]. This research highlights the use of serial passages to study host-pathogen relationships and has increased the understanding of how *C. neoformans* may interact differently with *Galleria* hemocytes compared to human and mouse macrophages.

## 4. Materials and Methods

### 4.1. Ethical Statement

The J774.16 cell line was obtained from Dr. Arturo Casadevall. The RAW264.7 cell line was provided by the American Type Culture Collection, Manassas, VA, USA.

### 4.2. Passages

*C. neoformans* serotype A strain H99W was grown in yeast peptone dextrose (YPD) broth from frozen stock at 37 °C for two–three days and then washed three times with phosphate-buffered saline (PBS) and counted. H99W cells (1 × 10^3^ in 5 µL) were injected into the last left proleg of 15 larvae of the wax worm, *Galleria mellonella* and then larvae were incubated at 37 °C for three days. After three days, larvae were rinsed in 100% ethanol and the last left proleg was cut with scissors. Hemolymph was collected from each larva, diluted and serial dilutions were plated for CFU on YPD agar. Non-diluted hemolymph from each larva was pooled and counted. Penicillin/Streptomycin (10,000 U/mL, Invitrogen, catalog #15140122, Carlsbad, CA, USA) was added to the pooled hemolymph and 1 × 10^3^ cells in 5 µL were injected into 15 larvae for the next passage.

Passages were continued until 100 generations of *C. neoformans* had occurred within *G. mellonella*. The doubling time was calculated based on the CFU of the initial inoculum and the average CFU from the 15 larvae from each passage using the following equation: Time infected*(1/Ln(Final CFU/Initial CFU)). The number of generations was calculated by dividing the time infected by the calculated doubling time for each passage.

### 4.3. In Vitro Growth Curves

Cultures of H99S, H99W and Pl5 were started from frozen stock and grown in YPD for 24 h. Cells were washed with PBS three times, counted using a hemocytometer, and diluted to 5000 cells/mL for each strain. These cells were then added to hemolymph extracted from healthy *Galleria*, YPD, minimal media, or a solution of 1:1 ratio of minimal media:hemolymph. To prevent melanization, all media containing hemolymph also contained 3 mg of 1-phenly-2-thiourea (PTU) crystals. Since PTU crystals may affect growth, cells were grown in all medias containing hemolymph with and without crystals. Cultures were then placed in multi-well plates and growth was measured for 24 h using the BioScreen C growth curve machine (Growth Curves USA, Piscataway, NJ, USA) and absorbance at 600 nm. The growth curves were repeated three times. Due to the non-orbital rotation of the machine and to avoid settling of the cells, cells were manually re-suspended after the first 8 h and then every 10 h for the duration of the study. Doubling times were calculated using the following formula: Time × (0.693/(Ln × (final OD/initial OD))). This experiment was repeated three times.

### 4.4. In Vivo Galleria Fungal Burden

Cultures of H99S, H99W and Pl5 were started from frozen stock and grown in YPD until log-phase. Cells were washed with PBS three times and counted. Five µL of each cell suspension (2000 cells) was injected into the last pro-leg of fifteen *Galleria* larvae for each strain for each time point. Hemolymph was collected from remaining larvae at days 1, 3 and 5. Serial dilutions of hemolymph were plated on YPD plates for 24 h and then the most appropriate dilution was plated on an additional YPD plate. Plates were incubated for two days at 37 °C and colonies counted to determine fungal burden. Fungal burden was normalized to the number of larvae collected at each day. This experiment was repeated three times.

### 4.5. Capsule Size

Capsule size in vitro was measured as described in [32,33]. This experiment was repeated three times.

### 4.6. GXM Release

To determine if there was a difference in the release of capsular GXM into the medium, the capsules of all strains were induced in Dulbecco’s modified Eagle medium (DMEM) (as for measuring capsule size). The next day, a capture enzyme-linked immunosorbent assay was used to determine the concentration of GXM in the supernatant, as described previously [34]. This experiment was repeated three times.

### 4.7. Mouse Macrophage Death and C. neoformans Intracellular Replication

To determine the susceptibility of macrophage-like J774.16 or RAW264.7 cells to killing by *C. neoformans* and to measure how well strains H99W and P15 replicated in macrophages, strains were incubated in a 2:1 multiplicity of infection with macrophage-like J774.16 or RAW264.7 cells in DMEM for 1 h at 37 °C + 5% CO_2_ (four wells per strain), washed with PBS, resuspended with DMEM and incubated for a further 18 h at 37 °C + 5% CO_2_, as described in [35]. The next day, cells were resuspended in 200 µL warm PBS containing 10 µg/mL propidium iodide and imaged as described in [35].

To measure *C. neoformans* intracellular replication, the saved media from the killing experiment (above) was combined with washes containing intracellular *C. neoformans,* diluted, and plated on YPD, as described in [35].

### 4.8. Isolation and Culture of Human Monocytes

Venous blood of healthy male and female volunteers was collected in accordance with the guidelines and approval of the Wright Center for Graduate Medical Education Institutional Review Board, Scranton, PA, USA. All blood donors were informed of the goals of the study and agreed by written consent prior to blood donation. Monocytes were isolated and cultured, as described in [35].

### 4.9. Human Macrophage Death and C. neoformans Intracellular Replication

To determine the susceptibility of human ex vivo macrophages to killing by *C. neoformans* and to measure how well strains H99W and P15 survived in human macrophages, macrophages were seeded into a 96-well plate (four wells per *C. neoformans* isolate) in Roswell Park Memorial Institute (RPMI)-1640 media containing 10% human serum, and 100 ng/mL lipopolysaccharide at a density of 2 × 10^4^ macrophages and incubated overnight at 37 °C with 5% CO_2_. Macrophage death and *C. neoformans* intracellular replication were determined as described in Section 4.7 above and [35]. 

### 4.10. Resistance to Hydrogen Peroxide

H99W and P15 were grown from frozen stocks in YPD at 37 °C for 2–3 days, washed three times with water and counted. To determine resistance to hydrogen peroxide and oxidative stress, strains were tested as described [29]. Briefly, 2 × 10^3^ cells were incubated with 0 or 0.5 mM hydrogen peroxide (Fisher, catalog #H325-100) for 3 or 6 h at 37 °C. At each time point, 100 µL of each sample was plated on YPD and grown at 37 °C for 2 days to determine CFU. Each condition was done in triplicate and the experiment was repeated twice. To determine how the induction of capsule affected resistance to hydrogen peroxide, capsule was induced as described above. After 18 h at 37 °C + 5% CO_2_, cells were collected, washed three times with water and resistance to hydrogen peroxide was tested. This experiment was repeated twice.

### 4.11. Resistance to Nitrosative Stress

H99W and P15 were grown from frozen stocks in YPD at 37 °C for two–three days, washed three times with minimal media and counted. To determine resistance to nitrosative stress, strains were tested as described in [36]. Briefly, 2 × 10^3^ cells were incubated in minimal media alone or with 0.5 mM sodium nitrate (Sigma, catalog #S5506) and 25 mM succinic acid (Sigma, catalog #S9512) for 3 or 6 h at 37 °C. At each time point, 100 (3 h) or 50 (6 h) µL of each sample was plated on YPD and grown at 37 °C for 2 days to determine CFU. Each condition was done in triplicate and the experiment was repeated three times.

### 4.12. Microarray Analysis

The experimental design and the data for the microarray have been deposited in NCBI’s Gene Expression Omnibus [37] and are accessible through GEO Series accession number GSE59582. *C. neoformans* strains H99W and P15 were grown in YPD from frozen stock at 37 °C for two–three days and then washed three times with PBS and counted. H99W or P15 (1 ×10^3^ cells in 5 µL) were injected into the last left proleg of 15 larvae of *G. mellonella* for each strain. After three days of incubation at 37 °C, larvae were rinsed in 100% ethanol (Sigma, catalog #E7023) and the last left proleg was cut with scissors. Hemolymph was collected from each larva and pooled by strain. The pooled hemolymph was centrifuged to pellet the cryptococcal cells, the hemolymph was removed and > 1 × 10^7^ cells were used to extract RNA using the RNeasy Kit (catalog # 75142, Qiagen, Hilden, Germany) with removal of genomic DNA (Message Clean Kit, catalog # M601, GenHunter, Nashville, TN, USA). Two different pools of RNA were analyzed at Washington University, using the *C. neoformans* JEC21 genomic microarray, which was developed by the Cryptococcus Community Microarray Consortium with financial support from individual researchers and the Burroughs Wellcome Fund (http://genome.wustl.edu/services/microarray/cryptococcus_neoformans), as described in [38].

### 4.13. STRING Analysis

Protein interaction networks among the 400 differentially expressed genes identified by microarray analysis were investigated using STRING (ELIXIR, Cambridgeshire, UK, version 11.0). Four hundred genes were used as input and 325 genes were identified within the database. To visualize the interactions from the dataset as a whole, single nodes were hidden and the remaining nodes clustered using MCL, a Markov clustering algorithm embedded within STRING with the inflation parameter set to 3.

### 4.14. qRT-PCR

cDNA was made from both strains H99W and P15 from the two pools of RNA isolated from *G. mellonella* and used for the microarray (Quantitech Reverse Transcription kit, catalog #205311, Qiagen). qRT-PCR was performed using SYBR Green (Applied Biosystems Foster City, CA, USA, catalog #4309155), cDNA, and primer in an ABI PRISM^®^ 7900HT Sequence Detection System (Applied Biosystems, Foster City, CA, USA). Each cDNA was amplified in quadruplicate, normalized with actin or glycerol-3-phosphate dehydrogenase and the fold change was determined [39]. Fold change for P15 was relative to H99W. Real-time qRT-PCR was repeated twice.

### 4.15. Hemocyte Hydrogen Peroxide Production

H99W, P15 and *C. albicans* (ATCC 90028) cultures were started from frozen stock and grown in YPD at 37 °C for 24 h (log-phase) prior to infection. Cells were washed with insect physiological saline (IPS), counted on a hemocytometer and diluted. Ten larvae per strain were infected with 2000 cells of H99W and P15 and 10,000 cells of *C. albicans* by injecting 5 µL of each cell suspension into the last pro-leg of each larvae. For the experiments using the GXM shedding mutants’ *liv7Δ* and *ima1Δ,* 10 larvae each were infected with 2000 cells of each strain. After 24 h of incubation, hemocytes were isolated from hemolymph by clipping a terminal leg and the hemolymph was removed by squeezing. Approximately 3 mg of PTU crystals were added to 1 mL of IPS to create a 1:200 dilution of PTU solution. One hundred microliters of the PTU solution was added to the extracted hemolymph from each strain infection to prevent melanization. Hemocytes were collected by centrifugation for 5 min at 400× *g*, counted with Trypan Blue to differentiate between dead and living hemocytes and resuspended in IPS. Cells were used immediately in the ThermoFisher Scientific Hydrogen Peroxide Assay Kit (ThermoFisher Scientific, Waltham, MA, USA) to determine the relative levels of H_2_O_2_ in hemocytes [40]. The assay was conducted by creating a 50 µM Amplex Red, 1 U/mL of Horse Radish Peroxidase (HRP) solution. Assays were performed in triplicate in a 96-well black plate. Each assay used 1000 cells per well in a total volume of 120 μL for each well. A SpectroMax M5 plate reader (Molecular Devices, San Jose, CA, USA) was used to monitor florescence every 2 min for 12–20 min with excitation at 560 nm and detection at 590 nm. All assays were normalized to cell counts for each strain. This experiment was repeated three times.

### 4.16. Histology

Two larvae were infected with 3000 cells of either H99W, P15, or H99S and incubated for two and five days at 37 °C. Larvae were then fixed in 4% paraformaldehyde at 4 °C for at least two days, bisected, sectioned, and stained with hematoxylin and eosin at the Translational Pathology Shared Resource of Vanderbilt University Medical Center. Images of each larva were captured using an Olympus BX60 microscope (Olympus Corporation, Tokyo, Japan) and the 10× objective. Nodule number and size were counted/measured using the center-most section of each larva.

### 4.17. Statistics

The in vivo *Galleria* fungal burden data was analyzed using analysis of variance (ANOVA) with simple contrasts. Capsule size and amount of GXM shed was analyzed using ANOVA. Intracellular replication of *C. neoformans* and macrophage killing were analyzed using the nonparametric Wilcoxon Rank Sums test, while resistance to hydrogen peroxide was analyzed using ANOVA with simple contrasts. The production of hydrogen peroxide by hemocytes was analyzed by linear regression and Tukey’s HSD for all pairwise comparisons. The number of nodules was analyzed using a chi-square test and differences in nodule size were analyzed using the nonparametric Wilcoxon Rank Sums test.

## Figures and Tables

**Table 1:** In vitro doubling times. The length of time (h) that it took for the adapted P15 strain, the parent strain and H99S to double their cell numbers in *Galleria* hemolymph.

Strain	Experiment 1	Experiment 2	Experiment 3
H99S	3.49	7.26	10.53
P15	2.14	2.03	2.50
H99W	3.16	3.30	0.95

## Supplementary Materials

The following are available online at https://www.mdpi.com/2076-0817/9/9/732/s1, Table S1: All genes showing gene expression changes greater than 2-fold in either direction between P15 and H99W. Highlighted genes were tested using qRT-PCR; Table S2. List of genes tested using qRT-PCR.
