# Supplementary material for: Serial Passage of Cryptococcus neoformans in Galleria mellonella Results in Increased Capsule and Intracellular Replication in Hemocytes, but Not Increased Resistance to Hydrogen Peroxide
Source: Pathogens. 2020 Sep 5;9(9):732. doi: 10.3390/pathogens9090732 (PMC7559301; doi:10.3390/pathogens9090732)
Supplement: Supplementary file 1 [file pathogens-09-00732-s001.zip › Table S1.docx]

Table 2. All genes showing gene expression changes greater than 2-fold in either direction between P15 and H99W. Highlighted genes were tested using qRT-PCR.

| **Locus ID** | **Gene ID** | **Description** | **GO/ Pathway Description** | **Classification** | **Average Ratio (P15/H99W)** |
| --- | --- | --- | --- | --- | --- |
| 184.m04746 | CNL04160 | Retrotransposon nucleocapsid protein, putative | DNA-directed DNA polymerase activity; RNA binding; RNA-directed DNA polymerase activity; peptidase activity; protein binding; ribonuclease activity | Cell cycle | 5.06 |
| 184.m04884 | CNL05670 | Hypothetical protein | DNA binding; Chromatin assembly/disassembly; nuclear nucleosome | Cell cycle | 4.28 |
| 176.m02374 | CNK02390 | Hypothetical protein | Tubulin binding; tubulin complex assembly | Cell cycle | 2.70 |
| 167.m03479 | CNE03330 | Ribonucleoside-diphosphate reductase large chain, putative | Ribonucleoside-diphosphate reductase activity; DNA replication | Cell cycle | 2.62 |
| 1662.seq.067 | CNAG_01736 | Predicted protein | DASH complex domain; microtubule binding; mitosis | Cell cycle | 2.61 |
| CNAG_04328 | CNI02210 | Ubiquitin-conjugating enzyme e2-18 | Mitotic spindle elongation; cell cycle | Cell cycle | 2.59 |
| 186.m04054 | CNB03040 | Single-stranded DNA binding protein 12k chain, putative | DNA replication; homologous recombination; mismatch repair | Cell cycle | 2.55 |
| CNAG_06402 | CNN01280 | Conserved hypothetical protein | Homologous recombination; proteosome | Cell cycle | 2.36 |
| 183.m01696 | CNN01280 | Hypothetical protein | Homologous recombination; proteosome | Cell cycle | 2.25 |
| CNAG_03835 | CNBB2440 | Predicted protein | DASH complex domain; microtubule binding; mitosis | Cell cycle | 2.25 |
| 1712.seq.180 | CNBB2440 | Hypothetical protein | DASH complex domain; microtubule binding; mitosis | Cell cycle | 2.22 |
| 1712.seq.158 | CNB03040 | Hypothetical protein | ssDNA binding protein; DNA replication; homologous recombination | Cell cycle | 2.21 |
| CNAG_05107 | CNL05150 | Gim complex component GIM3 | Tubulin binding; tubulin complex assembly; prefoldin complex | Cell cycle | 2.08 |
| 164.m02815 | CNM01830 | Origin recognition complex subunit 4 | DNA replication origin binding; DNA dependent DNA replication | Cell cycle | 2.01 |
| 181.m08527 | CNA07850 | Hypothetical protein | Microfilament motor activity; budding; endocytosis; cytokinesis | Cell wall | 2.50 |
| 179.m00540 | CNC06280 | Conserved hypothetical protein | Calcium ion binding; Phosphatidylinositol signaling; cell budding; mitosis | Cell wall | 2.39 |
| 163.m06205 | CND02720 | Conserved hypothetical protein | Structural molecule activity; actin cortical patch assembly | Cell wall | 2.35 |
| CNAG_00902 | CND00430 | Conserved hypothetical protein | Transmembrane protein | Cell wall | 2.35 |
| 180.m00134 | CNF02990 | Hypothetical protein | Phosphate/phosphoenolpyruvate translocator | Cell wall | 2.33 |
| CNAG_04240 | CNI03810 | Conserved hypothetical protein | Microtubule production | Cell wall | 2.13 |
| 181.m08020 | CNA02590 | Mitochondrion protein, putative | Mitochondria | Energy | 2.50 |
| CNAG_02529 | CNE00370 | Conserved hypothetical protein | Complex1_LYR-like domain; Fe-S cluster biogenesis in mitochondria | Energy | 2.12 |
| 179.m00147 | CNC01610 | Hypothetical protein | DNA binding; Chromatin assembly/disassembly; nuclear nucleosome | Housekeeping | 3.94 |
| 181.m08832 | CNA07460 | Hypothetical protein | Complex I intermediate associated protein 30 domain | Housekeeping | 3.06 |
| 179.m00020 | CNC00190 | Hydrolase, putative | Hydrolase activity; nucleotide binding; nucleotide metabolism | Housekeeping | 2.66 |
| CNAG_03069 | CNC00190 | Hydrolase | Hydrolase activity; nucleotide binding; nucleotide metabolism | Housekeeping | 2.30 |
| 163.m06267 | CND03220 | mRNA processing-related protein, putative | mRNA processing; spliceosome | Housekeeping | 2.22 |
| 164.m02090 | CNM00840 | Expressed protein | Shwachman-Bodian-Diamond syndrome (SBDS) protein domain; RNA metabolism | Housekeeping | 2.12 |
| CNAG_02425 | CNE01320 | Conserved hypothetical protein | Nucleotidyl transferase domain | Housekeeping | 2.11 |
| CNAG_01891 | CNK02320 | RAD57 protein | P-loop containing Nucleoside Triphosphate Hydrolase domain | Housekeeping | 2.08 |
| 179.m00519 | CNC05950 | Phosphoribosyl-ATP diphosphatase, putative | Phosphoribosyl-ATP diphosphatase activity; purine metabolsim; cytoplasm; nucleus | Metabolism | 5.69 |
| CNAG_07146 | CNAG_07744 | 1-phosphatidylinositol 4-kinase | Inositol Phosphate Metabolism | Metabolism | 5.16 |
| 181.m08852 | CNA03190 | Hypothetical protein | Apolipoprotein O domain | Metabolism | 4.95 |
| CNAG_04392 | CNI01560 | Sterol-binding protein | SCP-2 sterol transfer family | Metabolism | 3.29 |
| 163.m06514 | CND04630 | Conserved hypothetical protein | Nitrogen metabolism; Cyanase C terminal domain | Metabolism | 3.04 |
| CNAG_06694 | CNF03210 | Transthyretin family protein | Purine metabolism | Metabolism | 2.96 |
| 163.m06513 | CND04610 | Hypothetical protein | Histidine phosphatase domain | Metabolism | 2.90 |
| 184.m04617 | CNH02830 | 2-nitropropane dioxygenase, putative | Nitrogen metabolism | Metabolism | 2.86 |
| 184.m04662 | CNH03280 | Isocitrate lyase, putative | Isocitrate lyase activity; glyoxylate cycle | Metabolism | 2.73 |
| 167.m03228 | CNE00710 | Mannitol-1-phosphate dehydrogenase, putative | Alcohol dehydrogenase activity; fermentation; fatty acid metabolism | Metabolism | 2.73 |
| CNAG_06140 | CNM01420 | Long-chain fatty acid transporter | Fatty-acyl-CoA binding; fatty acid metabolism | Metabolism | 2.56 |
| CNAG_03679 | CNBB3950 | Conserved hypothetical protein | Acylphosphatase domain | Metabolism | 2.48 |
| 164.m02127 | CNM01420 | Long-chain fatty acid transporter, putative | Acyl-CoA binding; long-chain fatty acid transporter activity; fatty acid metabolism | Metabolism | 2.46 |
| 167.m03357 | CNE02100 | Uracil phosphoribosyltransferase 1, putative | Uracil phosphoribosyltransferase activity; pyrimidine metabolism | Metabolism | 2.44 |
| CNAG_07765 | CNI01580 | Hypothetical protein | Pyridoxine 5'-phosphate (PNP) oxidase-like domain | Metabolism | 2.18 |
| 1661.seq.116 | CNC01090 | Hypothetical protein | Ubiquinol-cytochrome C chaperone | Metabolism | 2.18 |
| 179.m00442 | CNC05150 | Conserved hypothetical protein | Cytochrome c oxidase subunit VIb | Metabolism | 2.17 |
| 163.m06527 | CND04470 | Expressed protein | Pyridoxine 5'-phosphate (PNP) oxidase-like domain | Metabolism | 2.12 |
| 1712.seq.006 | CNBB3950 | Hypothetical protein | Acylphosphatase domain | Metabolism | 2.06 |
| 1761.seq.054 | CNBK2960 | Hypothetical protein | CBS domain | Metabolism | 2.05 |
| CNAG_00002 | CGB_K0100W | Predicted protein | Amidohydrolase domain | Metabolism | 2.04 |
| CNAG_01047 | CND01760 | Conserved hypothetical protein | D-amino acid oxidase domain; amino acid transport and metabolism | Metabolism | 2.01 |
| 167.m03273 | CNE01180 | Conserved hypothetical protein | YcaC related amidohydrolase; urease | Pathogenicity | 2.72 |
| 180.m00213 | CNF04430 | Antiphagocytic protein, putative |  | Pathogenicity | 2.71 |
| 1631.seq.002 | CNAG_07457 | Hypothetical protein | Antibiotic biosynthesis domain | Pathogenicity | 2.39 |
| CNAG_06574 | CNF04430 | Antiphagocytic protein 1 |  | Pathogenicity | 2.16 |
| 185.m02487 | CNJ01400 | Conserved hypothetical protein | Baculoviral inhibition of apoptosis protein repeat domain | Pathogenicity | 2.15 |
| 181.m08535 | CNA07930 | Microtubule binding protein, putative | Microtubule binding; autophagy; protein targeting to vacuole | Protein Degradation | 2.80 |
| 186.m03748 | CNB03140 | Ubiquitin-protein ligase, putative | Protein binding & processing; ubiquitin-protein ligase activity; cell cyle | Protein Degradation | 2.79 |
| 1743.seq.162 | CNBJ0680 | Hypothetical protein | Protein processing in ER | Protein Degradation | 2.41 |
| CNAG_02112 | CNE04250 | Conserved hypothetical protein | Ubiquitin-conjugating enzyme E2 domain; ubiquitin mediated proteolysis | Protein Degradation | 2.38 |
| 167.m03565 | CNE04250 | Hypothetical protein | Ubiquitin-conjugating enzyme E2 domain; ubiquitin mediated proteolysis | Protein Degradation | 2.32 |
| CNAG_03837 | CNB03250 | Protein-L-isoaspartate O-methyltransferase | Posttranslational modification; protein turnover | Protein Integrity | 2.63 |
| 186.m03760 | CNB03250 | Conserved hypothetical protein | Protein-L-isoaspartate carboxylmethyltransferase; posttranslational modification | Protein Integrity | 2.45 |
| 179.m00098 | CNC01010 | Cation transport-related protein, putative | Cation transport; plasma membrane | Secretion | 3.60 |
| CNC01010 | CNC01010 | Cation transport-related protein, putative | Cation transport; plasma membrane | Secretion | 3.54 |
| CNAG_03952 | CNB04400 | Conserved hypothetical protein | COPI associated protein | Secretion | 3.20 |
| 186.m03876 | CNB04400 | Expressed protein | COPI associated protein | Secretion | 2.97 |
| 181.m08844 | CNA00580 | Conserved hypothetical protein | Protein transporter activity; cotranslational protein targeting to membrane | Secretion | 2.78 |
| CNAG_00067 | CNA00580 | Conserved hypothetical protein | Protein transporter activity; cotranslational protein targeting to membrane | Secretion | 2.77 |
| 162.m02784 | CNI02440 | Conserved hypothetical protein | Got1/Sft2-like family domain; vesicular transport | Secretion | 2.71 |
| 184.m04455 | CNH01380 | Conserved hypothetical protein | Protein binding; endocytosis; late endosome to vacuole transport | Secretion | 2.42 |
| CNAG_05266 | CNH03640 | Membrane protein | Transporter Activity | Secretion | 2.37 |
| 177.m03057 | CNG02120 | Calcium ion binding protein, putative | Calcium ion binding; membrane | Secretion | 2.30 |
| 1701.seq.063 | CNA00580 | Hypothetical protein | Protein transporter activity; cotranslational protein targeting to membrane | Secretion | 2.17 |
| 186.m03727 | CNB02980 | Calcium:hydrogen antiporter, putative | Calcium ion transmembrane transporter activity; calcium:hydrogen antiporter activity | Secretion | 2.16 |
| CNAG_01872 | CNK02120 | Copper ion transporter | Copper ion transmembrane transporter activity; fungal vacuole membrane | Secretion | 2.10 |
| 181.m07945 | CNA01710 | Retrograde transport, endosome to Golgi-related protein | Vacuolar sorting protein; endosome | Secretion | 2.10 |
| CNAG_02313 | CNE02330 | Conserved hypothetical protein | Modifier of rudimentary (Mod(r)) protein domain; vacuolar protein sorting | Secretion | 2.05 |
| 179.m00031 | CNC00310 | Hmp1 protein, putative | CsbD-like domain | Stress response | 4.35 |
| CNAG_01446 | CND05600 | Hypothetical protein | Heat shock protein activity; response to heat, oxidative stress | Stress response | 2.43 |
| 186.m03741 | CNB03070 | Hypothetical protein | Endopeptidase activity; response to stress; proteosome core complex | Stress response | 2.41 |
| 185.m02558 | CNJ02120 | Hypothetical protein | Stress response domain | Stress response | 2.38 |
| 183.m01603 | CNN00220 | Glutathione peroxidase, putative | Glutathione peroxidase activity; response to oxidative stress | Stress response | 2.34 |
| 163.m06340 | CND05600 | Conserved hypothetical protein | Heat shock protein activity; response to heat, oxidative stress | Stress response | 2.30 |
| 177.m03264 | CNG04220 | Heat shock protein (glucose and lipid-regulated protein), putative | Heat shock protein activity; response to heat, oxidative stress | Stress response | 2.26 |
| CNAG_03984 |  | Conserved hypothetical protein | Rhodanese Homology Domain | Stress response | 2.16 |
| 176.m02396 | CNK02620 | ATP-dependent protein binding protein | ATP-dependent protein binding; protein ubiquitination; response to stress | Stress response | 2.08 |
| 167.m03214 | CNE00580 | Glutathione peroxidase | Glutathione peroxidase activity; response to oxidative stress | Stress response | 2.08 |
| 186.m03921 | CNB04860 | Protein hob3 | Cytoskeletal protein binding; endocytosis; membrane raft; response to stress | Stress response | 2.03 |
| 1731.seq.094 | CNBI2080 | Hypothetical protein | DNA directed RNA polymerase M; transcription elongation factor | Transcription | 2.59 |
| 177.m02897 | CNG00450 | Transcription initiation factor iia small chain | General RNA polymerase II transcription factor activity; transcription initiation | Transcription | 2.09 |
| 163.m06259 | CND03250 | Conserved hypothetical protein | Transcription corepressor activity; negative regulation of transcription | Transcription | 2.04 |
| 1682.seq.142 | CNF02950 | Conserved hypothetical protein | DNA binding transcription factor activity; spliceosome | Transcription | 2.01 |
| CNAG_04387 | CNI01600 | Pre-mRNA splicing factor | mRNA splicing; spliceosome; small nuclear ribonucleoprotein complex | Translation | 3.51 |
| 185.m02692 | CNJ03360 | Hypothetical Protein | Nuclear mRNA splicing; spliceosome; small nuclear ribonucleoprotein complex | Translation | 3.20 |
| 163.m06345 | CND05700 | Conserved hypothetical protein | Structural constituent of ribosome; translation | Translation | 3.04 |
| CNAG_04155 | CNI02920 | Small nuclear ribonucleoprotein E | Nuclear mRNA splicing; spliceosome; small nuclear ribonucleoprotein complex | Translation | 2.93 |
| CNAG_04914 | CNJ03360 | Conserved hypothetical protein | Nuclear mRNA splicing; spliceosome; small nuclear ribonucleoprotein complex | Translation | 2.76 |
| 181.m07899 | CNA01220 | Cyclophilin, putative | Poly(A) binding; regulation of translational initiation | Translation | 2.69 |
| 162.m02822 | CNI02920 | Small nuclear ribonucleoprotein E, putative | Nuclear mRNA splicing; spliceosome; small nuclear ribonucleoprotein complex | Translation | 2.67 |
| 181.m08538 | CNA07960 | Conserved hypothetical protein | Structural constituent of ribosome; translation | Translation | 2.62 |
| CNAG_02898 | CNC05100 | RNA binding protein | mRNA binding and export from the nucleus; spiceosome | Translation | 2.38 |
| 1663.seq.129 | CNC04440 | Ribosomal chaperone, putative | Ribosomal chaperone activity; structural constituent of ribosome; translation | Translation | 2.28 |
| 162.m02773 | CNI02170 | 60s ribosomal protein l31, mitochondrial precursor (yml31), putative | Structural constituent of ribosome; translation | Translation | 2.23 |
| 167.m03536 | CNE03910 | Expressed protein | Ribosomal L28 domain | Translation | 2.08 |
| CNAG_02811 | CNC04290 | Hypothetical protein | Structural constituent of ribosome; translation | Translation | 2.06 |
| 186.m03715 | CNB02870 | Mitochondrial 40s ribosomal protein | Structural constituent of ribosome; translation | Translation | 2.05 |
| 179.m00459 | CNC05320 | Mitochondrial 60s ribosomal protein l38 (yml38) | Structural constituent of ribosome; translation | Translation | 2.05 |
| Stratagene oligo 1 |  | Stratagene oligo 1 |  | Unknown | 11.50 |
| Stratagene oligo 3 |  | Stratagene oligo 3 |  | Unknown | 8.62 |
| Stratagene oligo 2 |  | Stratagene oligo 2 |  | Unknown | 6.44 |
| CNAG_02271 | CNAG_07642 | Hypothetical Protein |  | Unknown | 5.31 |
| CNAG_06516 | CNN02390 | Conserved hypothetical protein |  | Unknown | 4.11 |
| Stratagene oligo 4 |  | Stratagene oligo 4 |  | Unknown | 3.70 |
| 181.m08838 | CNA00410 | Expressed protein |  | Unknown | 3.59 |
| 181.m08820 | CNA06450 | Hypothetical protein |  | Unknown | 3.59 |
| CNAG_01493 | CNBC0300 | Conserved hypothetical protein |  | Unknown | 3.46 |
| Stratagene oligo 6 |  | Stratagene oligo 6 |  | Unknown | 3.38 |
| 184.m04789 | CNL04630 | Hypothetical protein |  | Unknown | 3.27 |
| CNAG_01735 | CNC02400 | Conserved hypothetical protein |  | Unknown | 3.26 |
| 180.m00024 | CNF00450 | Hypothetical protein |  | Unknown | 3.26 |
| 179.m00220 | CNC02400 | Expressed protein |  | Unknown | 3.17 |
| 1681.seq.049 | CNF00380 | Hypothetical protein |  | Unknown | 3.16 |
| CNAG_05162 | CNL04630 | Predicted protein |  | Unknown | 3.14 |
| 1751.seq.098 | CNAG_07939 | Predicted protein |  | Unknown | 3.11 |
| CNAG_00679 | CNA06570 | Conserved hypothetical protein |  | Unknown | 2.89 |
| Stratagene oligo 7 |  | Stratagene oligo 7 |  | Unknown | 2.88 |
| CNAG_05167 | CNL04580 | Conserved hypothetical protein |  | Unknown | 2.85 |
| 183.m01613 | CNN00330 | Expressed protein |  | Unknown | 2.82 |
| 163.m06465 | CND03770 | Hypothetical protein |  | Unknown | 2.77 |
| 184.m04698 | CNH03650 | Hypothetical protein |  | Unknown | 2.77 |
| CNAG_06297 | CNN00330 | Conserved hypothetical protein |  | Unknown | 2.73 |
| RPL39_grubii_MATa_RPL39\|grubii |  | RPL39_grubii_MATa_RPL39\|grubii |  | Unknown | 2.68 |
| 184.m05164 | CNL04580 | Expressed protein |  | Unknown | 2.67 |
| CNAG_04366 |  | Predicted protein |  | Unknown | 2.64 |
| 180.m00127 | CNF02910 | CNF02910;expressed protein; |  | Unknown | 2.59 |
| CNAG_05930 | CNF00450 | Conserved hypothetical protein |  | Unknown | 2.58 |
| Stratagene oligo 5 |  | Stratagene oligo 5 |  | Unknown | 2.49 |
| 1762.seq.125 | CNAG_01942 | Hypothetical protein |  | Unknown | 2.47 |
| 167.m05771 | CNE00400 | Expressed protein |  | Unknown | 2.44 |
| CNAG_03937 |  | Predicted protein |  | Unknown | 2.39 |
| 185.m02664 | CNJ03120 | Hypothetical protein |  | Unknown | 2.38 |
| 1664.seq.202 | CNAG_01493 | Hypothetical protein |  | Unknown | 2.32 |
| CNAG_03337 | CNG02420 | Conserved hypothetical protein |  | Unknown | 2.29 |
| 181.m08403 | CNA06570 | Expressed protein |  | Unknown | 2.28 |
| CNAG_02591 |  | Hypothetical protein |  | Unknown | 2.28 |
| 184.m05115 | CNH02940 | Hypothetical protein |  | Unknown | 2.27 |
| 162.m04528 | CNI01610 | Conserved hypothetical protein |  | Unknown | 2.27 |
| CNAG_06274 |  | Hypothetical protein |  | Unknown | 2.27 |
| 180.m02941 | CNF01920 | Hypothetical protein | Transposase domain | Unknown | 2.26 |
| CNAG_04105 |  | Hypothetical protein |  | Unknown | 2.25 |
| CNAG_03933 | CNB04210 | Transmembrane protein 167 |  | Unknown | 2.20 |
| CNAG_00075 | CNA00650 | Conserved hypothetical protein |  | Unknown | 2.19 |
| 1772.seq.032 | CNBG2680 | Hypothetical protein |  | Unknown | 2.19 |
| 181.m08432 | CNA06900 | Conserved hypothetical protein |  | Unknown | 2.18 |
| CNBG2650 | CNBG2650 | Hypothetical protein | Calcium binding domain | Unknown | 2.16 |
| 177.m03083 | CNG02420 | Hypothetical protein |  | Unknown | 2.16 |
| CNAG_01215 |  | Conserved hypothetical protein |  | Unknown | 2.15 |
| CNAG_00985 | CND01130 | Conserved hypothetical protein |  | Unknown | 2.13 |
| CNAG_04300 | CNI02500 | Conserved hypothetical protein |  | Unknown | 2.10 |
| 1712.seq.015 | CNBB3860 | Hypothetical protein |  | Unknown | 2.10 |
| 176.m02205 | CNK00650 | Hypothetical protein |  | Unknown | 2.09 |
| 163.m03852 | CND01770 | Expressed protein |  | Unknown | 2.08 |
| CNAG_00859 | CNE05280 | Conserved hypothetical protein |  | Unknown | 2.06 |
| 164.m02001 | CNM00270 | Expressed protein |  | Unknown | 2.05 |
| 180.m02965 | CNF00380 | Hypothetical protein |  | Unknown | 2.04 |
| 162.m02805 | CNI02270 | Expressed protein |  | Unknown | 2.03 |
| CNAG_00536 | CNA05180 | Conserved hypothetical protein |  | Unknown | 2.02 |
| CNAG_04396 | CNI01520 | Aycl012 |  | Unknown | 2.02 |
| 186.m04071 | CNB04210 | Hypothetical protein |  | Unknown | 2.01 |
| 1713.seq.088 | CNAG_03937 | Hypothetical protein |  | Unknown | 2.01 |
| 163.m03773 | CND00110 | ATP-dependent DNA helicase | ATP-dependent DNA helicase activity; DNA unwinding involved in replication; meiotic chromosome segregation | Cell cycle | -2.82 |
| 167.m03678 | CNE00510 | DNA repair protein rad16 | DNA-dependent ATPase activity; nucleotide-excision repair, DNA damage recognition | Cell cycle | -2.61 |
| 181.m08109 | CNA03560 | Conserved hypothetical protein | Helicase activity | Cell cycle | -2.53 |
| 1742.seq.056 | CNF03140 | Retrotransposon nucleocapsid protein | DNA-directed DNA polymerase activity; RNA binding; RNA-directed DNA polymerase activity; peptidase activity; protein binding; ribonuclease activity | Cell cycle | -2.48 |
| 1743.seq.159 | CNAG_04850 | Hypothetical protein | Exonuclease domain | Cell cycle | -2.45 |
| 163.m06502 | CND04390 | Hypothetical protein | Co-chaperone activity; post-chaperonin tubulin folding pathway | Cell cycle | -2.44 |
| 163.m06574 | CND00680 | Peptidase | SUMO-specific protease activity; cysteine-type peptidase activity; chromosome condensation; mitotic cell cycle spindle assembly | Cell cycle | -2.42 |
| 181.m08320 | CNA05680 | Actin monomer binding protein | Actin monomer binding; actin polymerization; bud site selection; response to osmotic stress | Cell cycle | -2.39 |
| CNBB1880 | CNB03850 | Hypothetical protein | p21-C-terminal region-binding domain | Cell cycle | -2.36 |
| 184.m04776 | CNL04490 | Conserved hypothetical protein | DNA-directed DNA polymerase activity; DNA repair; DNA-dependent DNA replication | Cell cycle | -2.34 |
| 167.m05797 | CNE00970 | Conserved hypothetical protein | ATPase activity; mitotic chromosome condensation | Cell cycle | -2.33 |
| 1711.seq.083 |  | Hypothetical protein | MATa pheramone | Cell cycle | -2.31 |
| AAN01358 |  | MATa grubii | MATa pheramone | Cell cycle | -2.30 |
| 181.m08390 | CNA06400 | Chromatin remodeling-related protein | Chromatin remodeling | Cell cycle | -2.26 |
| 181.m08407 | CNA06610 | Kinesin | Microtubule motor activity; microtubule depolymerization | Cell cycle | -2.26 |
| 180.m00139 | CNF03140 | Retrotransposon nucleocapsid protein | DNA-directed DNA polymerase activity; RNA binding; RNA-directed DNA polymerase activity; peptidase activity; protein binding; ribonuclease activity | Cell cycle | -2.25 |
| 186.m04109 | CNB03920 | Conserved hypothetical protein | Regulator of chromosome condensation domain; Alpha-tubulin suppressor | Cell cycle | -2.24 |
| 181.m07825 | CNA00400 | Histone deacetylation-related protein | NAD-dependent & NAD-independent histone deacetylase activity; negative regulation of meiosis | Cell cycle | -2.23 |
| 181.m08183 | CNA04250 | Cyclin | Cyclin-dependent protein kinase regulator activity; cyclin-dependent protein kinase holoenzyme complex | Cell cycle | -2.22 |
| 186.m03488 | CNB00740 | Kinesin | Microtubule motor activity; microtubule depolymerization | Cell cycle | -2.21 |
| 177.m03389 | CNG02090 | Conserved hypothetical protein | DNA binding; DNA-dependent DNA replication | Cell cycle | -2.16 |
| 184.m05153 | CNL03950 | Hypothetical protein | Telomerase activating protein Est1; DNA/RNA binding domain | Cell cycle | -2.11 |
| CNBJ0350 | CNBJ0350 | Hypothetical protein | Myosin heavy chain domain | Cell cycle | -2.10 |
| 181.m08755 | CNA03370 | Expressed protein | Chromosome segregation protein SMC | Cell cycle | -2.10 |
| CNBI0320 | CNBI0320 | Hypothetical protein | Chromosome segregation protein SMC | Cell cycle | -2.10 |
| 177.m03175 | CNG03350 | Hypothetical protein | Ribonucleotide reductase inhibitor | Cell cycle | -2.09 |
| CNAG_02669 | CNBF1830 | Hypothetical protein | Integrase core domain; psq DNA binding domain | Cell cycle | -2.02 |
| CNM01240 | CNM01240 | Hypothetical protein | DNA-dependent ATPase activity; chromatin assembly | Cell cycle | -2.02 |
| 186.m03877 | CNB04410 | Cell growth and/or maintenance-related protein | yrdC domain | Cell cycle | -2.01 |
| 181.m08700 | CNA01070 | C-14 sterol reductase | Ergosterol biosynthesis | Cell wall | -2.58 |
| 183.m01693 | CNN01260 | Plasma membrane H(+)-ATPase 1 | Hydrogen-exporting ATPase activity; oxidative phosphorylation; regulation of pH | Cell wall | -2.58 |
| 1773.seq.045 | CNAG_03169 | Hypothetical protein | RTA1-like protein | Cell wall | -2.44 |
| 179.m00054 | CNC00560 | Hypothetical protein | Large tegument protein UL36 domain | Cell wall | -2.42 |
| CNBB1610 | CNB04130 | Nitrilase-like protein | Hydrolase activity | Cell wall | -2.42 |
| 180.m00238 | CNF04830 | Hydroxymethylglutaryl-CoA reductase (NADPH) | Hydroxymethylglutaryl-CoA reductase (NADPH) activity; ergosterol biosynthesis | Cell wall | -2.32 |
| CNAG_05070 | CNL05500 | Sulfite reductase beta subunit | Sulfite reductase (NADPH) activity; sulfur reduction and metabolism; cell wall organization | Cell wall | -2.30 |
| 184.m04400 | CNH00800 | GPI anchor biosynthesis-related protein | GPI anchor biosynthesis; membrane | Cell wall | -2.21 |
| 186.m04078 | CNB04600 | Uridine transporter | Uridine transmembrane transporter activity; uridine transport; plasma membrane | Cell wall | -2.20 |
| 183.m01822 | CNN00260 | Sugar transporter | Galactose & glucose transmembrane transporter activity; extracellular carbohydrate transport | Cell wall | -2.20 |
| 1772.seq.162 | CNA04790 | Hypothetical protein | CCC1 domain; transmembrane protein | Cell wall | -2.18 |
| 186.m03837 | CNB04020 | AE016780 membrane protein | Multidrug transporter activity; response to toxin; membrane | Cell wall | -2.17 |
| CNAG_00507 |  | Nitrilase | Breaks carbon-nitrogen bonds; ergosterol biosynthesis domain | Cell wall | -2.14 |
| 184.m05011 | CNH02310 | Hypothetical protein | 7 transmembrane receptor (Secretin family) domain | Cell wall | -2.11 |
| CND04140 | CND04140 | Hypothetical protein | Inositol-pentakisphosphate 2-kinase domain; mRNA export; endocytosis | Cell wall | -2.09 |
| 181.m08519 | CNA07770 | Conserved hypothetical protein | Glucan 1,3-beta-glucosidase activity; fungal cell wall | Cell wall | -2.09 |
| 164.m02145 | CNM01860 | Conserved hypothetical protein | Ion channel regulatory protein | Cell wall | -2.08 |
| 162.m04552 | CNI02740 | Conserved hypothetical protein | Signal transducer activity; cell-cell adhesion; filamentous growth; plasma membrane | Cell wall | -2.08 |
| 1681.seq.018 | CNF00160 | Rho small monomeric GTPase | Rho small monomeric GTPase activity; signal transducer activity; endo/exocytosis; psuedohyphal growth | Cell wall | -2.07 |
| 183.m01597 | CNN00150 | Alpha-glucoside:hydrogen symporter | Alpha-glucoside:hydrogen symporter activity; alpha-glucoside transport | Cell wall | -2.06 |
| 180.m00240 | CNF04870 | Conserved hypothetical protein | Adaptin N terminal region domain; clathrin-coated pits & vesicles | Cell wall | -2.05 |
| 163.m03838 | CND00790 | Glucan 1,3-beta-glucosidase | Glucan 1,3-beta-glucosidase activity; fungal cell wall | Cell wall | -2.05 |
| 177.m03042 | CNG01940 | Mitochondrial inner membrane protein | Inner mitochondrial membrane organization | Energy | -2.56 |
| CNAG_05875 | CNF00970 | Cytochrome c heme lyase | Holocytochrome-c synthase activity; cytochrome c-heme linkage | Energy | -2.53 |
| 179.m05178 | CNC01100 | Hypothetical protein | Cytochrome oxidase complex assembly protein 1 domain | Energy | -2.18 |
| 1771.seq.029 | CNAG_03576 | Cytochrome C assembly protein |  | Energy | -2.13 |
| CNAG_00161 | CNA01490 | Auxin-induced protein | Aldo-keto reductase domain | Energy | -2.03 |
| 180.m00250 | CNF00290 | Nucleoside-diphosphatase | Nucleoside-diphosphatase activity; protein glycosylation | Housekeeping | -2.52 |
| 180.m00417 | CNF03660 | GPI-anchor transamidase | GPI-anchor transamidase activity; integral to ER membrane | Housekeeping | -2.43 |
| 163.m02751 | CND02670 | Endoplasmic reticulum protein | Integral membrane protein domain | Housekeeping | -2.12 |
| 184.m05045 | CNH03800 | Telomere maintenance protein | RNA binding; telomeric DNA binding; mRNA export from nucleus | Housekeeping | -2.09 |
| 177.m03207 | CNG03670 | Adenylate cyclase | Adenylate cyclase activity; signal transduction; meiosis | Housekeeping | -2.07 |
| 176.m02496 | CNK00240 | Deadenylation-dependent decapping-related protein | Enzyme activator activity; hydrolase activity; mRNA binding & catabolism; RNA degradation | Housekeeping | -2.02 |
| 185.m02426 | CNJ00800 | Conserved hypothetical protein | Succinate-CoA ligase (ADP-forming) activity; succinyl-CoA metabolism; TCA cycle | Metabolism | -3.35 |
| 167.m03575 | CNE04360 | Fatty-acid synthase complex protein | 3-oxoacyl-[acyl-carrier-protein] reductase and synthase activity; holo-[acyl-carrier-protein] synthase activity; fatty acid biosynthesis | Metabolism | -2.73 |
| 181.m08240 | CNA04870 | Conserved hypothetical protein | Peptidyl-prolyl cis-trans isomerase activity | Metabolism | -2.68 |
| 183.m01673 | CNN01010 | Malate dehydrogenase (oxaloacetate-decarboxylating) | Malate dehydrogenase (oxaloacetate-decarboxylating) activity; pyruvate metabolism; cellular amino acid metabolism | Metabolism | -2.59 |
| 164.m01361 | CNM02510 | Conserved hypothetical protein | Glycine/D-amino acid oxidase domain | Metabolism | -2.58 |
| 177.m03122 | CNG02820 | Protein phosphatase regulator | Protein phosphatase regulator activity; protein dephosphorylation | Metabolism | -2.49 |
| 186.m03656 | CNB02270 | Peroxisome targeting sequence binding protein | Peroxisome matrix targeting signal-1 binding; peroxisome targeting sequence binding | Metabolism | -2.47 |
| 186.m04112 | CNB04010 | Hypothetical protein | Alpha/beta hydrolase domain | Metabolism | -2.47 |
| 181.m08254 | CNA04970 | Mannitol dehydrogenase | Alcohol dehydrogenase (NADP+) activity; alcohol metabolism | Metabolism | -2.44 |
| 162.m02869 | CNI03300 | Phosphoribosylformylglycinamidine synthase | Phosphoribosylformylglycinamidine synthase activity; purine biosynthesis and metabolism | Metabolism | -2.40 |
| 181.m08255 | CNA04980 | Peroxisome targeting signal receptor | Peroxisome matrix targeting signal-2 binding | Metabolism | -2.40 |
| 163.m06192 | CND02600 | Conserved hypothetical protein | Crotonase/Enoyl-Coenzyme A (CoA) hydratase domain | Metabolism | -2.40 |
| CNAG_06374 | CNN01010 | Malate dehydrogenase | Pyruvate metabolism; cellular amino acid metabolism | Metabolism | -2.39 |
| 1703.seq.100 | CNA04630 | Hypothetical protein | 4-diphosphocytidyl-2C-methyl-D-erythritol kinase | Metabolism | -2.35 |
| 179.m00287 | CNC03230 | Long-chain acyl-CoA synthetase | AMP binding; cytoplasm; peroxisome | Metabolism | -2.31 |
| 181.m07991 | CNA02260 | 8-amino-7-oxononanoatesynthase | 5-aminolevulinate synthase activity; biotin metabolism; heme biosynthesis | Metabolism | -2.31 |
| 162.m02903 | CNI03690 | 2,4-dienoyl-CoA reductase (NADPH) | 2,4-dienoyl-CoA reductase (NADPH) activity; ascospore formation; fatty acid catabolism | Metabolism | -2.29 |
| 163.m06434 | CND03650 | Conserved hypothetical protein | Enzyme activator activity; phospholipid metabolism; vacuole | Metabolism | -2.25 |
| CNAG_07746 | CNG00590 | Methylenetetrahydrofolate dehydrogenase (NADP) | Folic acid and derivative metabolism-related protein | Metabolism | -2.24 |
| 179.m00704 | CNC04500 | UDP-glucose:sterol glucosyltransferase | Sterol 3-beta-glucosyltransferase activity; sterol metabolism | Metabolism | -2.16 |
| 181.m08219 | CNA04660 | Tricarboxylic acid cycle-related protein | 2-oxoglutarate metabolism; TCA cycle | Metabolism | -2.16 |
| CNAG_01952 | CNK02910 | Aryl-alcohol dehydrogenase | Aryl-alcohol dehydrogenase (NAD+) activity; cellular aldehyde metabolism | Metabolism | -2.12 |
| 179.m00075 | CNC00800 | Biotin-[acetyl-CoA-carboxylase] ligase | Biotin-[acetyl-CoA,methylcrotonoyl-CoA-carboxylase] ligase activity; biotin-[methylmalonyl-CoA-carboxytransferase] ligase activity; biotin-[propionyl-CoA-carboxylase (ATP-hydrolyzing)] ligase activity; biotin metabolism; protein modification | Metabolism | -2.11 |
| 163.m06395 | CND06170 | Oxidoreductase | Oxidoreductase activity | Metabolism | -2.10 |
| 163.m03769 | CND00030 | Maltose O-acetyltransferase | Acetyltransferase activity; maltose O-acetyltransferase activity | Metabolism | -2.08 |
| 167.m03400 | CNE02620 | Branched-chain alpha-keto acid dehydrogenase E1-alpha subunit | Pyruvate dehydrogenase (acetyl-transferring) activity; pyruvate metabolism | Metabolism | -2.06 |
| 185.m02399 | CNJ00540 | L-aminoadipate-semialdehyde dehydrogenase | L-aminoadipate-semialdehyde dehydrogenase activity; lysine biosynthesis | Metabolism | -2.01 |
| 186.m03873 | CNB04360 | 4-nitrophenylphosphatase | 4-nitrophenylphosphatase activity; alkaline phosphatase activity; protein dephosphorylation | Metabolism | -2.01 |
| 185.m02509 | CNJ01650 | Extracellular elastinolytic metalloproteinase precursor | Fungalysin metallopeptidase domain | Pathogenicity | -4.34 |
| CNAG_04215 | CNI03560 | Sulfate adenylyltransferase | Sulfate adenylyltransferase (ATP) activity; melanin biosynthesis | Pathogenicity | -2.54 |
| 163.m06590 | CND01030 | Conserved hypothetical protein | Metallo-beta-lactamase domain | Pathogenicity | -2.31 |
| 183.m01674 | CNN01020 | Vacuolar membrane protein | Golgi to endosome to vacuole transport; fungal type vacuole | Pathogenicity | -2.28 |
| CNAG_07937 | CNN01530 | O-acetyltransferase | Transferase activity; capsule biosynthesis; membrane | Pathogenicity | -2.15 |
| 163.m06210 | CND02630 | Hypothetical protein | Cytochrome-c peroxidase activity; response to oxidative stress; mitochondria | Pathogenicity | -2.11 |
| 186.m03632 | CNB02040 | Efflux protein | Drug resistance transporter | Pathogenicity | -2.10 |
| 186.m03674 | CNB02490 | Conserved hypothetical protein | Short chain dehydrogenase domain; THN_reductase-like_SDR_c; melanin biosynthesis | Pathogenicity | -2.07 |
| 180.m00255 | CNF00080 | Membrane protein, putative | Proteasome | Protein Degradation | -2.54 |
| 180.m00067 | CNF01270 | Conserved hypothetical protein | Ubiquitin-mediated proteolysis; protein ubiquitination | Protein Degradation | -2.18 |
| 181.m07950 | CNA01760 | Hypothetical protein | Ubiquitin-specific protease activity; protein deubiquitination | Protein Integrity | -2.59 |
| CNAG_00180 | CNA01690 | Carboxyl-terminal proteinase | Ubiquitin-specific protease activity; protein deubiquitination | Protein Integrity | -2.16 |
| 162.m02970 | CNI00640 | Conserved hypothetical protein | Ubiquitin-specific protease activity; protein deubiquitination | Protein Integrity | -2.15 |
| CNAG_00074 | CNA00640 | Integral to plasma membrane protein | Plasma-membrane choline transporter domain | Secretion | -3.17 |
| 184.m04335 | CNH00110 | Hypothetical protein | Transporter activity; D-galactonate transporter domain | Secretion | -2.70 |
| 177.m02990 | CNG01400 | Conserved hypothetical protein | ER to Golgi vesicle-mediated transport; protein complex assembly | Secretion | -2.49 |
| 179.m00538 | CNC06250 | Conserved hypothetical protein | ER to Golgi vesicle-mediated transport; protein complex assembly | Secretion | -2.49 |
| 163.m06448 | CND03840 | Vacuole fusion, non-autophagic-related protein | Vacuole fusion, non-autophagic; Polyphosphate polymerase domain of VTC4 | Secretion | -2.39 |
| 180.m00360 | CNF02310 | Late endosome to vacuole transport-related protein | Endocytosis; late endosome to vacuole transport | Secretion | -2.37 |
| 181.m07962 | CNA01870 | Gamma-adaptin | Clathrin binding; vesicle-mediated transport; adaptin domain | Secretion | -2.33 |
| 184.m05159 | CNL04600 | Expressed protein | Transcriptional regulator IP4 domain; membrane fusion protein domain | Secretion | -2.32 |
| 1642.seq.002 | CNG01170 | Hypothetical protein | Transporter Activity | Secretion | -2.18 |
| CNBF0100 | CNF04800 | Monocarboxylic acid transporter | Transporter Activity | Secretion | -2.12 |
| 167.m03699 | CNE02910 | Hexose transport-related protein | Fructose, galactose, glucose & mannose transmembrane transporter activity; plasma membrane | Secretion | -2.10 |
| 176.m02232 | CNK00910 | Tbc1 domain family protein | Rab GTPase activator activity; vesicle-mediated transport | Secretion | -2.06 |
| 181.m08452 | CNA07090 | ABC transporter | Xenobiotic-transporting ATPase activity; response to drug & oxygen; plasma membrane | Secretion | -2.04 |
| 184.m04496 | CNH01700 | Conserved hypothetical protein | Protein binding; Golgi to plasma membrane transport; exocytosis | Secretion | -2.03 |
| 1751.seq.003 | CNF04930 | Trahalose Transporter | Trehalose transmembrane transporter activity; Trehalose transport | Secretion | -2.01 |
| 185.m02534 | CNJ01890 | Conserved hypothetical protein | DENN domain | Signal Transduction | -2.47 |
| 163.m04797 | CND01520 | Hypothetical protein | Meprin and TRAF-C domain | Signal Transduction | -2.35 |
| 184.m04333 | CNH00090 | Hypothetical protein | Arrestin N terminal domain | Signal Transduction | -2.32 |
| 176.m02438 | CNK03060 | C2H2 zinc finger protein Zas1A | Fungal specific transcription factor domain | Signal Transduction | -2.30 |
| 180.m00044 | CNF00900 | Hypothetical protein | Transcription activator activity; regulation of carbohydrate metabolism | Signal Transduction | -2.30 |
| 163.m06364 | CND05760 | Ste11alpha protein | MAP kinase kinase kinase activity; pheromone-dependent signal transduction involved in conjugation with cellular fusion | Signal Transduction | -2.28 |
| 185.m02602 | CNJ02560 | Signal transducer | Rho GTPase activator activity; signal transducer activity; invasive growth in response to glucose limitation | Signal Transduction | -2.20 |
| CNAG_07675 | CNF01820 | Hypothetical protein | Ras GTPase activator | Signal Transduction | -2.19 |
| CNAG_03551 | CNG00500 | Predicted protein | Zinc finger domain; SPX domain | Signal Transduction | -2.14 |
| CNAG_03037 | CNC00480 | WDR8 protein |  | Signal Transduction | -2.12 |
| CNB03170 | CNB03170 | Hypothetical protein | Rgp1 domain; guanyl-nucleotide exchange factor | Signal Transduction | -2.12 |
| 184.m04517 | CNH01890 | Conserved hypothetical protein | Fusaric acid resistance protein domain | Stress response | -2.47 |
| 1662.seq.100 | CNC02670 | Metalloreductase | Ferric reductase binding domain; NADPH oxidase | Stress response | -2.37 |
| 183.m01598 | CNN00160 | Protein-histidine kinase | Osmosensor activity; protein histidine kinase activity; two-component sensor activity; response to hydrogen peroxide | Stress response | -2.35 |
| 176.m02580 | CNK02170 | Conserved hypothetical protein | ATP-dependent protein binding; protein ubiquitination; response to stress | Stress response | -2.14 |
| 181.m07928 | CNA01500 | Alternative oxidase 1 | Alternative oxidase activity; metabolism; pathogenesis; response to oxidative stress | Stress response | -2.04 |
| CNAG_00500 | CNA04830 | PP2Cp protein phosphotase | Protein dephosphorylation; response to osmotic stress | Stress response | -2.03 |
| CNAG_00520 | CNA05020 | Conserved hypothetical protein | Sequence-specific DNA binding transcription factor activity | Transcription | -3.24 |
| 185.m02610 | CNJ02660 | Transcriptional regulatory protein | DNA binding Transcription factor activity | Transcription | -2.70 |
| 180.m00282 | CNF00950 | Transcription/repair factor TFIIH subunit Tfb3 | General RNA polymerase II transcription factor activity; negative regulation of transcription from RNA polymerase II promoter during mitosis | Transcription | -2.58 |
| 186.m03709 | CNB02820 | Expressed protein | Putative transcriptional repressor domain | Transcription | -2.56 |
| 167.m03609 | CNE04710 | Helicase | ATPase activity; regulation of transcription from RNA polymerase II promoter | Transcription | -2.47 |
| 185.m02427 | CNJ00810 | RNA polymerase III transcription factor | RNA polymerase III transcription factor activity | Transcription | -2.43 |
| 1761.seq.060 | CNBN2030 | Hypothetical protein | RNA-dependent DNA polymerase; Integrase core domain | Transcription | -2.32 |
| 163.m06130 | CND02400 | Hypothetical protein | RNA polymerase II promoter domain; golgi-body localization domain | Transcription | -2.29 |
| 177.m03276 | CNG04340 | Transcription factor iiia | RNA polymerase III transcription factor activity | Transcription | -2.20 |
| 177.m03142 | CNG03040 | Conserved hypothetical protein | General RNA polymerase II transcription factor activity; regulation of transcription | Transcription | -2.15 |
| CNBK3340 | CNK00090 | Hypothetical protein | Homeodomain: DNA binding domain | Transcription | -2.14 |
| 185.m02547 | CNJ02030 | Nucleus protein | Fungal specific transcription factor domain; DNA binding domain | Transcription | -2.13 |
| CNBC2140 | CNC05030 | Hypothetical protein | dsRNA-specific ribonuclease | Transcription | -2.07 |
| 1641.seq.184 | CNAG_06163 | Hypothetical protein | Zn finger domain C2H2 | Transcription | -2.03 |
| 181.m08268 | CNA05100 | WD-repeat protein | General transcriptional repressor activity | Transcription | -2.03 |
| 184.m05169 | CNL05080 | Conserved hypothetical protein | DNA binding transcription factor activity; regulation of transcription | Transcription | -2.02 |
| 162.m02828 | CNI02700 | Hypothetical protein | GAL4-like Zn2Cys6 binuclear cluster DNA-binding domain; transcriptional regulation | Transcription | -2.01 |
| 181.m07989 | CNA02240 | Conserved hypothetical protein | tRNA dihydrouridine synthase activity; tRNA modification | Translation | -3.43 |
| 186.m03463 | CNB00500 | Hypothetical protein | mRNA binding; nuclear mRNA splicing | Translation | -2.54 |
| 181.m08362 | CNA06130 | Expressed protein | Urb2 domain | Translation | -2.51 |
| 185.m02392 | CNJ00490 | Eukaryotic translation initiation factor 2C 2 | Argonaute domain | Translation | -2.40 |
| CNAG_00386 | CNA03740 | Eukaryotic initiation factor 4F subunit P130 | RNA transport; translation initiation factor activity; ribosome | Translation | -2.36 |
| 163.m06511 | CND04580 | Chaperone | ATP binding; chaperone activity | Translation | -2.35 |
| 177.m03294 | CNG04580 | Hypothetical protein | Structural constituent of ribosome; mitochondrial large ribosomal subunit | Translation | -2.35 |
| 185.m02423 | CNJ00760 | Hypothetical protein | RNA binding domain | Translation | -2.32 |
| CNAG_00464 | CNA04430 | Pre-mRNA-splicing factor RSE1 | U2 snRNA binding; spliceosome assembly; nuclear mRNA splicing | Translation | -2.27 |
| 163.m06132 | CND02420 | Elongation factor 3 | ATPase activity; translation elongation factor activity | Translation | -2.26 |
| 179.m00699 | CNC03940 | PM-scl autoantigen | 3-5-exoribonuclease activity; RNA degradation | Translation | -2.20 |
| 167.m03417 | CNE02810 | tRNA binding protein | Ran GTPase binding; tRNA binding & export | Translation | -2.20 |
| 179.m00190 | CNC02040 | DNAj protein | Co-chaperone activity; protein folding; proteolysis | Translation | -2.16 |
| 184.m04610 | CNH02750 | Protein phosphatase PP2A0 B subunit gamma isoform | Protein dephosphorylation; translation; meiosis | Translation | -2.15 |
| 183.m01834 | CNN02200 | Ribonuclease H | 3-5 exonuclease activity; ribonuclease H activity; Ribosome biogenesis | Translation | -2.14 |
| 180.m00297 | CNF01250 | rRNA primary transcript binding protein | rRNA primary transcript binding; snoRNA binding; spliceosome | Translation | -2.14 |
| 181.m07907 | CNA01310 | Phosphoribosylamidoimidazole-succinocarboxamide synthase | Tyrosine-tRNA ligase activity; ribonucleotide synthase | Translation | -2.11 |
| 181.m08810 | CNA06150 | Cytoplasm protein | RNA binding domain | Translation | -2.09 |
| 181.m08090 | CNA03340 | Expressed protein | MED6 mediator sub complex component domain | Translation | -2.05 |
| Stratagene oligo 8 |  | Stratagene oligo 8 |  | Unknown | -10.29 |
| 1641.seq.170 | CNM01510 | Expressed protein |  | Unknown | -3.26 |
| 186.m03989 | CNB05430 | Expressed protein |  | Unknown | -2.81 |
| 1632.seq.066 | CNAG_01122 | Hypothetical protein |  | Unknown | -2.79 |
| 1663.seq.006 | CNAG_01841 | Hypothetical protein |  | Unknown | -2.73 |
| 185.m02504 | CNJ01600 | Expressed protein |  | Unknown | -2.65 |
| 1681.seq.020 | CND06350 | Hypothetical protein |  | Unknown | -2.63 |
| 184.m04890 | CNL05740 | Hypothetical protein |  | Unknown | -2.48 |
| 1704.seq.044 | CNA07680 | Expressed protein |  | Unknown | -2.48 |
| 185.m02732 | CNJ00630 | Hypothetical protein |  | Unknown | -2.47 |
| 184.m04710 | CNL03770 | Expressed protein |  | Unknown | -2.47 |
| 177.m03028 | CNG01790 | Hypothetical protein |  | Unknown | -2.44 |
| 163.m04784 | CND01410 | Hypothetical protein |  | Unknown | -2.43 |
| 185.m02531 | CNJ01860 | Hypothetical protein |  | Unknown | -2.40 |
| CNAG_03387 |  | Conserved hypothetical protein |  | Unknown | -2.39 |
| CNAG_00456 | CNA04360 | Conserved hypothetical protein |  | Unknown | -2.35 |
| 1641.seq.127 | CNAG_06105 | Hypothetical protein |  | Unknown | -2.33 |
| 186.m03945 | CNB05010 | Hypothetical protein |  | Unknown | -2.33 |
| 163.m04754 | CND00490 | Hypothetical protein |  | Unknown | -2.26 |
| 186.m03755 | CNB03200 | Hypothetical protein |  | Unknown | -2.25 |
| 167.m03427 | CNE02920 | Expressed protein |  | Unknown | -2.25 |
| 179.m00597 | CNC06960 | Hypothetical protein | Zinc finger, C3HC4 type domain; protein-protein interactions | Unknown | -2.24 |
| 179.m00378 | CNC04380 | Hypothetical protein |  | Unknown | -2.22 |
| CNAG_06159 | CNM01610 | Conserved hypothetical protein |  | Unknown | -2.22 |
| 186.m03946 | CNB05020 | Hypothetical protein |  | Unknown | -2.21 |
| 185.m02669 | CNJ03170 | Expressed protein |  | Unknown | -2.20 |
| gi\|7305154\|r |  | Hypoxanthine guanine phosphoribosyl transferase |  | Unknown | -2.20 |
| 185.m02382 | CNJ00370 | Expressed protein |  | Unknown | -2.19 |
| CNAG_04756 | CNJ01860 | Conserved hypothetical protein |  | Unknown | -2.19 |
| 1641.seq.028 | CNM00280 | Hypothetical protein |  | Unknown | -2.19 |
| 1741.seq.105 |  | Hypothetical protein |  | Unknown | -2.18 |
| 176.m02171 | CNK00340 | Hypothetical protein |  | Unknown | -2.18 |
| CNAG_02390 |  | Hypothetical protein |  | Unknown | -2.18 |
| 1641.seq.077 | CNM00660 | Hypothetical protein |  | Unknown | -2.18 |
| CNAG_05493 |  | Hypothetical protein |  | Unknown | -2.17 |
| CNAG_04626 | CNJ00630 | Conserved hypothetical protein |  | Unknown | -2.17 |
| 162.m03601 | CNI03800 | Hypothetical protein |  | Unknown | -2.16 |
| 177.m03286 | CNG04440 | Hypothetical protein |  | Unknown | -2.16 |
| 163.m06191 | CND02580 | Conserved hypothetical protein | Retinoic acid induced 16-like protein domain | Unknown | -2.16 |
| 1663.seq.036 | CNAG_02729 | Hypothetical protein |  | Unknown | -2.15 |
| 180.m00086 | CNF01850 | Expressed protein |  | Unknown | -2.15 |
| CNAG_04421 |  | Hypothetical protein |  | Unknown | -2.15 |
| 184.m05085 | CNH01670 | Expressed protein |  | Unknown | -2.15 |
| CNAG_00037 |  | Hypothetical protein |  | Unknown | -2.14 |
| 186.m03791 | CNB03490 | Hypothetical protein |  | Unknown | -2.13 |
| 179.m00662 | CNC02230 | Expressed protein |  | Unknown | -2.13 |
| 1701.seq.031 |  | Hypothetical protein |  | Unknown | -2.12 |
| CNAG_01193 | CND03170 | Conserved hypothetical protein |  | Unknown | -2.12 |
| CNAG_00702 | CNA06810 | Conserved hypothetical protein |  | Unknown | -2.11 |
| 1633.seq.162 | CNF04840 | Hypothetical protein |  | Unknown | -2.10 |
| CNAG_00079 | CNA00690 | Conserved hypothetical protein |  | Unknown | -2.10 |
| 1634.seq.053 | CNAG_07405 | Hypothetical protein |  | Unknown | -2.09 |
| 1681.seq.010 | CNE03210 | Hypothetical protein |  | Unknown | -2.09 |
| 177.m03442 | CNG02660 | Hypothetical protein |  | Unknown | -2.08 |
| 1671.seq.033 | CNBE0260 | Hypothetical protein |  | Unknown | -2.08 |
| CNAG_03119 |  | Predicted protein |  | Unknown | -2.08 |
| 177.m03293 | CNG04550 | Hypothetical protein |  | Unknown | -2.08 |
| 186.m03849 | CNB04100 | Hypothetical protein |  | Unknown | -2.07 |
| CNAG_05453 |  | Conserved hypothetical protein | Mating type alpha locus | Unknown | -2.07 |
| CNAG_01474 | CNC07050 | Conserved hypothetical protein |  | Unknown | -2.06 |
| 167.m05837 | CNE04330 | Hypothetical protein |  | Unknown | -2.06 |
| 186.m04035 | CNB01270 | Hypothetical protein |  | Unknown | -2.06 |
| 180.m02960 | CNF04840 | Expressed protein |  | Unknown | -2.06 |
| 180.m00413 | CNF03240 | Expressed protein |  | Unknown | -2.06 |
| CNAG_06164 | CNM01650 | Conserved hypothetical protein |  | Unknown | -2.06 |
| 177.m03090 | CNG02490 | Expressed protein |  | Unknown | -2.05 |
| CNAG_04736 | CNJ01660 | Conserved hypothetical protein |  | Unknown | -2.05 |
| 177.m03227 | CNG03890 | Hypothetical protein |  | Unknown | -2.05 |
| 1672.seq.162 | CNE03210 | Hypothetical protein |  | Unknown | -2.05 |
| 186.m04106 | CNB03570 | Hypothetical protein | Hepatocellular carcinoma-associated antigen 59 | Unknown | -2.04 |
| CNAG_07275 |  | Hypothetical protein |  | Unknown | -2.04 |
| CNAG_01032 | CND01640 | Conserved hypothetical protein |  | Unknown | -2.04 |
| CNAG_04223 |  | Predicted protein |  | Unknown | -2.04 |
| CNAG_00242 |  | Predicted protein |  | Unknown | -2.04 |
| CNAG_05719 | CNF02550 | Conserved hypothetical protein |  | Unknown | -2.03 |
| CNAG_06257 | CNA02120 | Hypothetical protein |  | Unknown | -2.03 |
| 1634.seq.069 |  | Hypothetical protein |  | Unknown | -2.02 |
| 181.m08568 | CNA08240 | Expressed protein |  | Unknown | -2.02 |
| CNAG_02537 |  | Predicted protein |  | Unknown | -2.02 |
| 176.m02172 | CNK00350 | Expressed protein |  | Unknown | -2.00 |
| CNBC1790 | CNBC1790 | Hypothetical protein |  | Unknown | -2.00 |
| CNAG_06618 | CNAG_07663 | Hypothetical protein |  | Unknown | -2.00 |
| MYO2_grubii_MATalpha_MYO2\|grubii |  | MYO2_grubii_MATalpha_MYO2\|grubii |  | Unknown | -2.00 |
