# Supplementary material for: Serial Passage of Cryptococcus neoformans in Galleria mellonella Results in Increased Capsule and Intracellular Replication in Hemocytes, but Not Increased Resistance to Hydrogen Peroxide
Source: Pathogens. 2020 Sep 5;9(9):732. doi: 10.3390/pathogens9090732 (PMC7559301; doi:10.3390/pathogens9090732)
Supplement: Supplementary file 1 [file pathogens-09-00732-s001.zip › Table S2.docx]

| Table S2. List of genes tested using qRT-PCR. | | |  |  |  |  |  |
| --- | --- | --- | --- | --- | --- | --- | --- |
| **Gene ID** | **Description** | **GO/ Pathway Description** | **Classification** | **Microarray FC** | **Q-PCR#1 FC** | **Q-PCR#2 FC** | **Validated?** |
| CNL05670 | Hypothetical protein | DNA binding; Chromatin assembly/disassembly; nuclear nucleosome | Cell cycle | 4.28 | 7.60 | 5.68 | Yes |
| CNI02210 | Ubiquitin-conjugating enzyme e2-18 | Mitotic spindle elongation; cell cycle | Cell cycle | 2.59 | 5.86 | 4.39 | Yes |
| CNC01610 | Hypothetical protein | DNA binding; Chromatin assembly/disassembly; nuclear nucleosome | Housekeeping | 3.94 | 2.68 | 1.94 | Yes |
| CND03220 | mRNA processing-related protein, putative | mRNA processing; spliceosome | Housekeeping | 2.22 | 5.98 | 4.05 | Yes |
| CNC05950 | Phosphoribosyl-ATP diphosphatase, putative | Phosphoribosyl-ATP diphosphatase activity; purine metabolsim; cytoplasm; nucleus | Metabolism | 5.69 | 9.65 | 7.19 | Yes |
| CNAG_07744 | 1-phosphatidylinositol 4-kinase | Inositol Phosphate Metabolism | Metabolism | 5.16 | No | -1.20 | No |
| CNM01420 | Long-chain fatty acid transporter | Fatty-acyl-CoA binding; fatty acid metabolism | Metabolism | 2.56 | 7.38 | 5.18 | Yes |
| CNF04430 | Antiphagocytic protein, putative |  | Pathogenicity | 2.71 | 5.74 | 4.27 | Yes |
| CNB03140 | Ubiquitin-protein ligase, putative | Protein binding & processing; ubiquitin-protein ligase activity; cell cyle | Protein Degradation | 2.79 | 6.59 | 5.02 | Yes |
| CNB03250 | Conserved hypothetical protein | Protein-L-isoaspartate carboxylmethyltransferase; posttranslational modification | Protein Integrity | 2.45 | 7.80 | 5.16 | Yes |
| CNK02120 | Copper ion transporter | Copper ion transmembrane transporter activity; fungal vacuole membrane | Secretion | 2.10 | 6.15 | 4.28 | Yes |
| CNC00310 | Hmp1 protein, putative | CsbD-like domain | Stress response | 4.35 | 8.47 | 6.34 | Yes |
| CND05600 | Hypothetical protein | Heat shock protein activity; response to heat, oxidative stress | Stress response | 2.43 | 5.91 | 3.93 | Yes |
| CNN00220 | Glutathione peroxidase, putative | Glutathione peroxidase activity; response to oxidative stress | Stress response | 2.34 | 6.78 | 4.57 | Yes |
| CNI01600 | Pre-mRNA splicing factor | mRNA splicing; spliceosome; small nuclear ribonucleoprotein complex | Translation | 3.51 | 10.85 | 8.19 | Yes |
| CNI02920 | Small nuclear ribonucleoprotein E | Nuclear mRNA splicing; spliceosome; small nuclear ribonucleoprotein complex | Translation | 2.93 | 6.64 | 4.93 | Yes |
| CNB02870 | Mitochondrial 40s ribosomal protein | Structural constituent of ribosome; translation | Translation | 2.05 | 5.65 | 3.82 | Yes |
| CNC05320 | Mitochondrial 60s ribosomal protein l38 (yml38) | Structural constituent of ribosome; translation | Translation | 2.05 | 3.75 | 2.48 | Yes |
| CNE00510 | DNA repair protein rad16 | DNA-dependent ATPase activity; nucleotide-excision repair, DNA damage recognition | Cell cycle | -2.61 | 2.04 | No | No |
| CNA04250 | Cyclin | Cyclin-dependent protein kinase regulator activity; cyclin-dependent protein kinase holoenzyme complex | Cell cycle | -2.22 | No | No | No |
| CNA01070 | C-14 sterol reductase | Ergosterol biosynthesis | Cell wall | -2.58 | -1.56 | -2.00 | Yes |
| CNN00260 | Sugar transporter | Galactose & glucose transmembrane transporter activity; extracellular carbohydrate transport | Cell wall | -2.20 | 2.28 | 2.03 | No |
| CNF00970 | Cytochrome c heme lyase | Holocytochrome-c synthase activity; cytochrome c-heme linkage | Energy | -2.53 | 1.23 | No | No |
| CNJ00800 | Conserved hypothetical protein | Succinate-CoA ligase (ADP-forming) activity; succinyl-CoA metabolism; TCA cycle | Metabolism | -3.35 | No | -1.14 | No |
| CNB02270 | Peroxisome targeting sequence binding protein | Peroxisome matrix targeting signal-1 binding; peroxisome targeting sequence binding | Metabolism | -2.47 | 1.58 | 1.26 | No |
| CNJ01650 | Extracellular elastinolytic metalloproteinase precursor | Fungalysin metallopeptidase domain | Pathogenicity | -4.34 | No | -1.79 | No |
| CNN01530 | O-acetyltransferase | Transferase activity; capsule biosynthesis; membrane | Pathogenicity | -2.15 | -1.41 | -1.49 | Yes |
| CND02630 | Hypothetical protein | Cytochrome-c peroxidase activity; response to oxidative stress; mitochondria | Pathogenicity | -2.11 | -3.57 | -4.00 | Yes |
| CNE02910 | Hexose transport-related protein | Fructose, galactose, glucose & mannose transmembrane transporter activity; plasma membrane | Secretion | -2.10 | 3.99 | 3.52 | No |
| CND05760 | Ste11alpha protein | MAP kinase kinase kinase activity; pheromone-dependent signal transduction involved in conjugation with cellular fusion | Signal Transduction | -2.28 | -1.22 | -1.52 | Yes |
| CNN00160 | Protein-histidine kinase | Osmosensor activity; protein histidine kinase activity; two-component sensor activity; response to hydrogen peroxide | Stress response | -2.35 | 1.42 | 1.34 | No |
| CNA01500 | Alternative oxidase 1 | Alternative oxidase activity; metabolism; pathogenesis; response to oxidative stress | Stress response | -2.04 | -1.12 | -1.25 | Yes |
| CNF00950 | Transcription/repair factor TFIIH subunit Tfb3 | General RNA polymerase II transcription factor activity; negative regulation of transcription from RNA polymerase II promoter during mitosis | Transcription | -2.58 | 1.36 | 1.11 | No |
| CNA02240 | Conserved hypothetical protein | tRNA dihydrouridine synthase activity; tRNA modification | Translation | -3.43 | 1.32 | -1.18 | No |
| CNA03740 | Eukaryotic initiation factor 4F subunit P130 | RNA transport; translation initiation factor activity; ribosome | Translation | -2.36 | -1.75 | -2.17 | Yes |
| CNA04430 | Pre-mRNA-splicing factor RSE1 | U2 snRNA binding; spliceosome assembly; nuclear mRNA splicing | Translation | -2.27 | 2.73 | 2.10 | No |
| FC= Fold Change. Each qPCR was done using either actin or GAPDH. If the gene expression levels did not match for both actin and GAPDH, "No" was entered for “Validation” of gene expression. | | | | | | | |
